# Metabolic profiling of adherence to diet, physical activity and body size recommendations for cancer prevention

**DOI:** 10.1038/s41598-018-34662-7

**Published:** 2018-11-02

**Authors:** Qianqian Gu, John J. Spinelli, Trevor B. J. Dummer, Treena E. McDonald, Steven C. Moore, Rachel A. Murphy

**Affiliations:** 1 School of Population and Public Health, University of British Columbia, Vancouver, BC Canada; 2 Cancer Control Research, BC Cancer Agency, Vancouver, BC Canada; 3 Division of Cancer Epidemiology & Genetics, National Cancer Institute, Bethesda, MD USA

## Abstract

Maintaining a healthy body weight, eating well and being physically active lowers cancer risk by 30%. However, the biology underlying these relationships is not well understood. We examined cross-sectional associations between metabolites and cancer preventive behaviors as well as the relevance to cancer-related pathways among 120 participants (50% men, mean BMI 26.6 kg/m^2^, mean age 54 years) with no history of smoking or cancer. Participants completed questionnaires, physical measurements and provided blood samples. Non-targeted nuclear magnetic resonance captured 223 metabolite measures. Factor analysis was performed separately for amino acid, fatty acid and lipoprotein groups. Multivariable-adjusted linear regression was used to evaluate associations between cancer preventive recommendations and metabolite-containing factors (p-value < 0.05, false discovery rate <0.20). An inflammation-related metabolite (glycoprotein acetylation) loaded strongly on a factor that was associated with excess adiposity (body fat ≥25% (men) or ≥30% (women) ß (SE) = 0.74 (0.18)) and not meeting physical activity recommendations (ß (SE) = 0.40 (0.20)). Insulin sensitivity-related metabolites including monounsaturated and polyunsaturated fats were lower among participants not meeting recommendations for adiposity, fruits and vegetables and physical activity while branched chain amino acids were higher. Cancer preventive behaviors were associated with complex metabolic signatures, including alterations in pathways known to be involved in cancer pathogenesis.

## Introduction

Cancer is a global problem and the burden of cancer is growing. In 2008, there were 12.7 million new cancer cases, which is projected to reach 22.2 million by 2030^1^. The increase in cancer incidence is a reflection of population growth, the aging population and change in the prevalence and distribution of risk factors. For example, the growing prevalence of obesity, a risk factor for numerous cancers, will lead to continuing increases in the burden of cancer in the coming decades^2^.

The International Agency for Research on Cancer has identified prevention as a critical component of cancer control^3^. Lifestyle is a key component of cancer prevention strategies, as maintaining a healthy body weight, eating a healthy diet and being physically active can prevent one-third of cancers^4^. The World Cancer Research Fund (WCRF) and American Institute for Cancer Research (AICR) have developed evidence-based guidelines on diet, body weight and physical activity for cancer prevention^5^. These guidelines emphasize: (1) achieving and maintaining a healthy weight throughout life - being as lean as possible throughout life without being underweight and avoiding excess weight gain; (2) adopting a physically active lifestyle including at least 150 minutes of moderate intensity or 75 minutes of vigorous intensity physical activity each week and limiting sedentary behavior; (3) eating a healthy diet with an emphasis on plant foods including eating at least 2.5 cups of fruits and vegetables each day, limiting consumption of red and processed meat, and choosing whole grains; and (4) limiting consumption of alcohol to no more than 1 drink per day for women or 2 drinks per day for men. Greater adherence to cancer preventive guidelines has been shown in multiple studies to reduce the risk of cancer development as well as mortality^6^. However, pathways linking engagement in preventive lifestyle behaviors and cancer are not fully understood.

Inflammation, insulin and the insulin like-growth factor axis, sex steroids, and specific dietary components or nutrients have all been hypothesized to play critical roles in the link between diet, body weight, physical activity and cancer development^7–10^. Identification of causative pathways is hampered by limitations of study designs whereby lifestyle factors are generally studied singularly (e.g. physical activity or body weight) and the number of pathways investigated is limited. Approaches that consider multiple lifestyle behaviors and provide information on multiple biological processes are needed to advance the field.

Metabolomics is the study of endogenous and exogenous small, low-molecular weight compounds that participate in metabolism. These small molecules (metabolites) include compounds such as lipids, sugars, amino acids, purines and bioactives involved in tissue signaling functions^11^. Advances in metabolomics profiling technology and processing have made it possible to analyze several hundred metabolites efficiently and precisely. Nuclear magnetic resonance (NMR)-based metabolomics can quantify several hundred metabolites in a single sample^12^. This approach thus has great potential for investigating lifestyle-cancer pathways. Therefore, the goals of this study were to characterize the association between metabolites and cancer preventive lifestyle behaviors and to determine the potential relevance of metabolite associations to pathways known to play a role in cancer development within a cross-sectional sample of adults from a population-based cohort. These behaviors have been studied individually in previous work in specific populations^13–16^, but to our knowledge, have not been investigated with respect to cancer prevention guidelines or in a Canadian cohort to determine whether associations are generalizable. Thus, a tertiary aim was to determine if findings in this middle-aged Canadian sample replicate prior evidence of metabolic associations with lifestyle behaviors.

## Results

Characteristics of the 120 participants are shown in Table 1. Half of participants were men, the mean age was 54 years, and 12% had no post-secondary education or less. The prevalence of participants meeting guidelines for healthy weight varied depending on the measurement used; 46% had healthy weight using BMI criteria, 64% had healthy weight using waist circumference criteria and 46% had healthy weight using body fat criteria. The majority of participants (61%) reported consuming fruits and vegetables at least 5 times per day, 91% reported having no more than 2 drinks per day (men) or no more than 1 drink per day (women), and 58% had at least 150 minutes of moderate activity or 75 minutes of vigorous physical activity per week.

**Table 1:** Characteristics of the sample of 120 participants from the BCGP.

Characteristic	
Age (years), Mean (SD)	53.6 (9.54)
Men, N (%)	60 (50%)
No post-secondary education, N (%)	14 (11.7)
BMI 18.5–24.9 kg/m^2^, N (%)	55 (45.8)
Waist circumference <102 cm (men) or <88 cm (women), N (%)	77 (64.2)
Body fat <25% (men) or <30% (women), N (%)	55 (45.8)
≥5 fruits and vegetables/d, N (%)	73 (60.8)
≥150 min moderate or ≥75 min vigorous physical activity/d, N (%)	70 (58.3)
≤2 drinks/d (men) or ≤1 drink/d (women), N (%)	109 (90.8)

**Figure 1:**
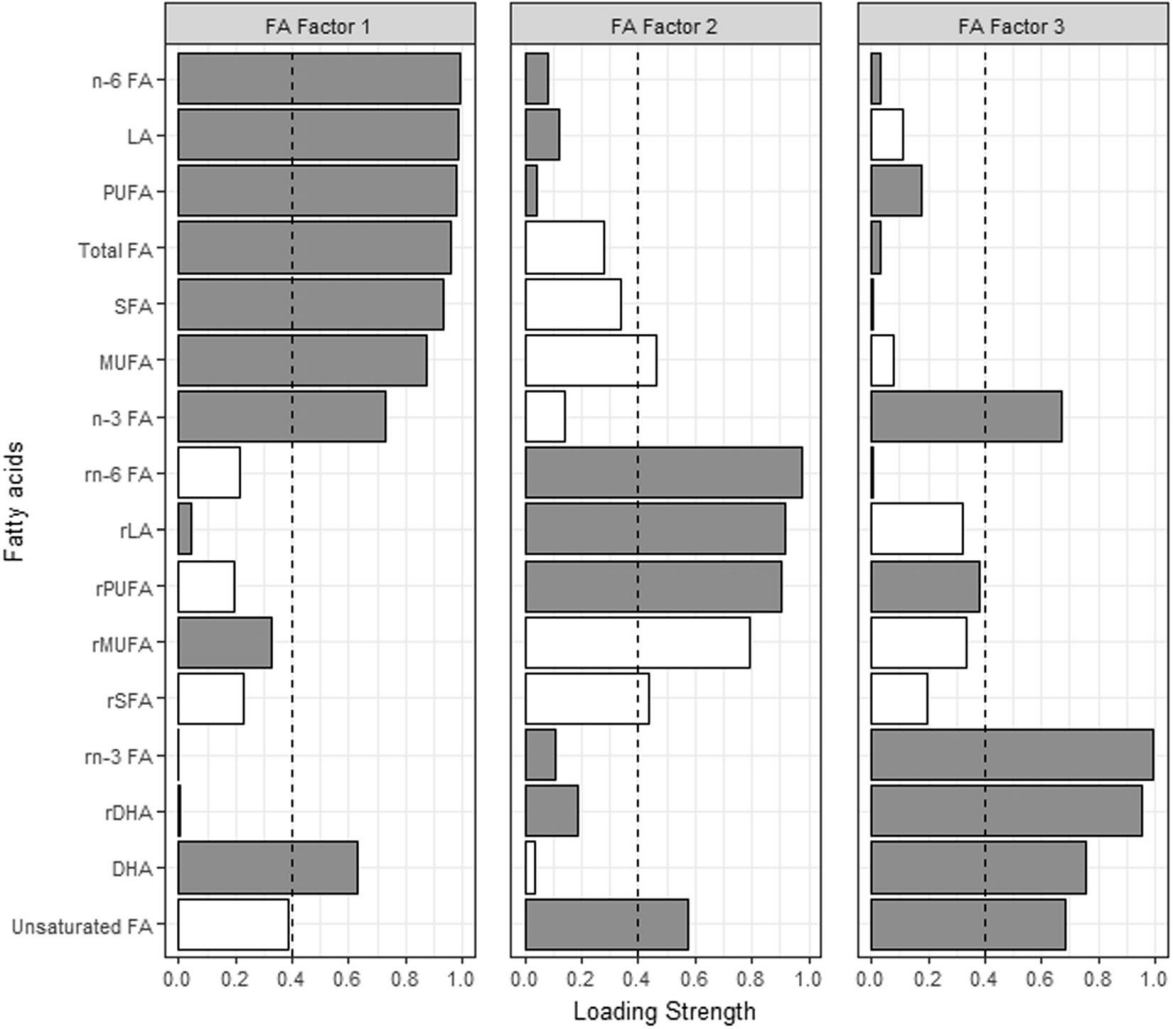
Factor loading plot for fatty acids metabolites. Grey bars indicate positive loadings, white bars indicate negative loadings, dashed lines indicate the threshold of 0.4 for identifying metabolites that load on a factor.

**Figure 2:**
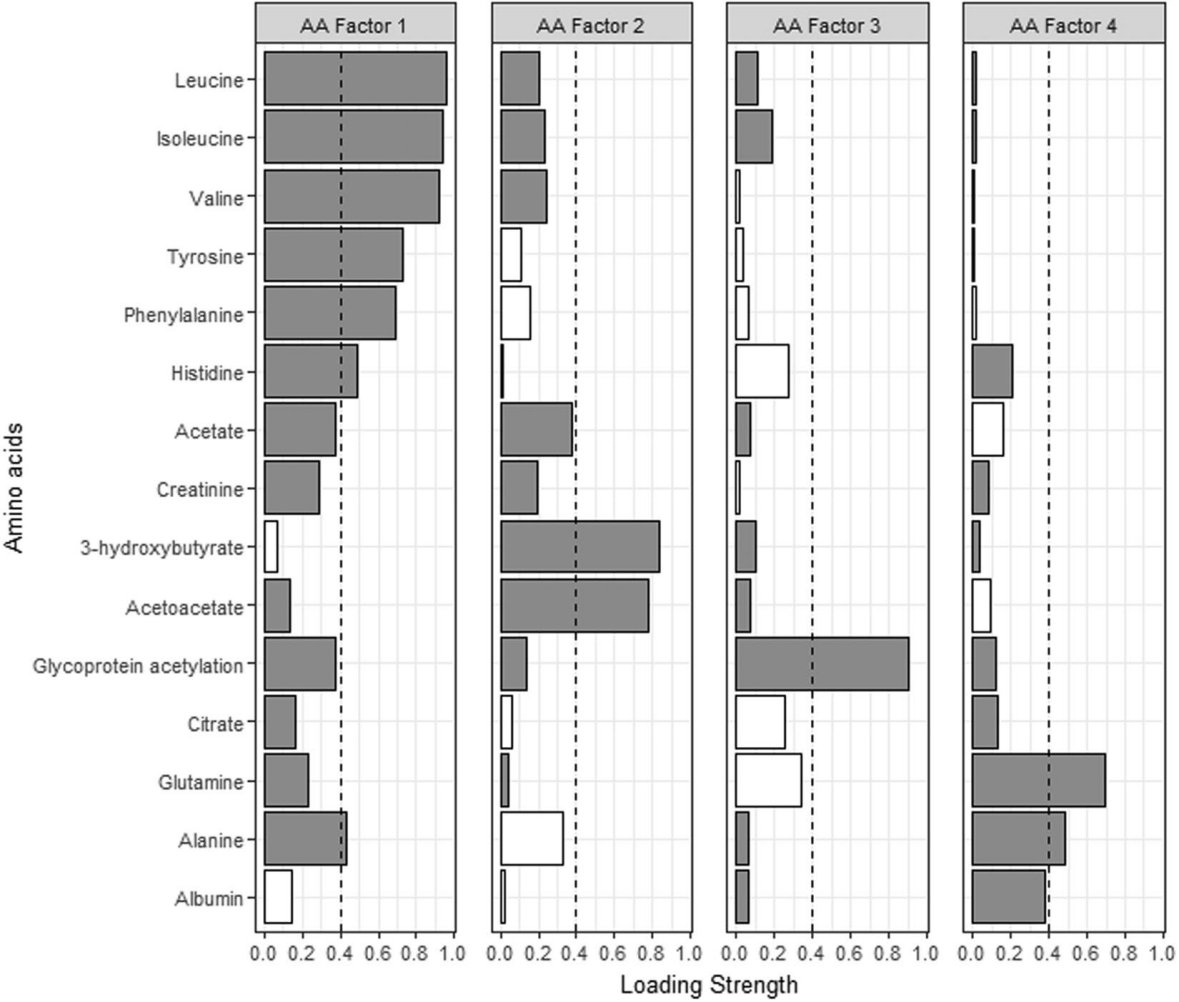
Factor loading plot for amino acid factors 1–4. Grey bars indicate positive loadings, white bars indicate negative loadings, dashed lines indicate the threshold of 0.4 for identifying metabolites that load on a factor. Abbreviations: FA; fatty acid, LA; linoleic acid, MUFA; monounsaturated fatty acids, AA; amino acid, C; cholesterol, CE; cholesterol ester, D; diameter, DHA; docosahexaenoic acid, PUFA; polyunsaturated fatty acids, SFA; saturated fatty acids, UnSat; estimated degree of unsaturation.

**Figure 3:**
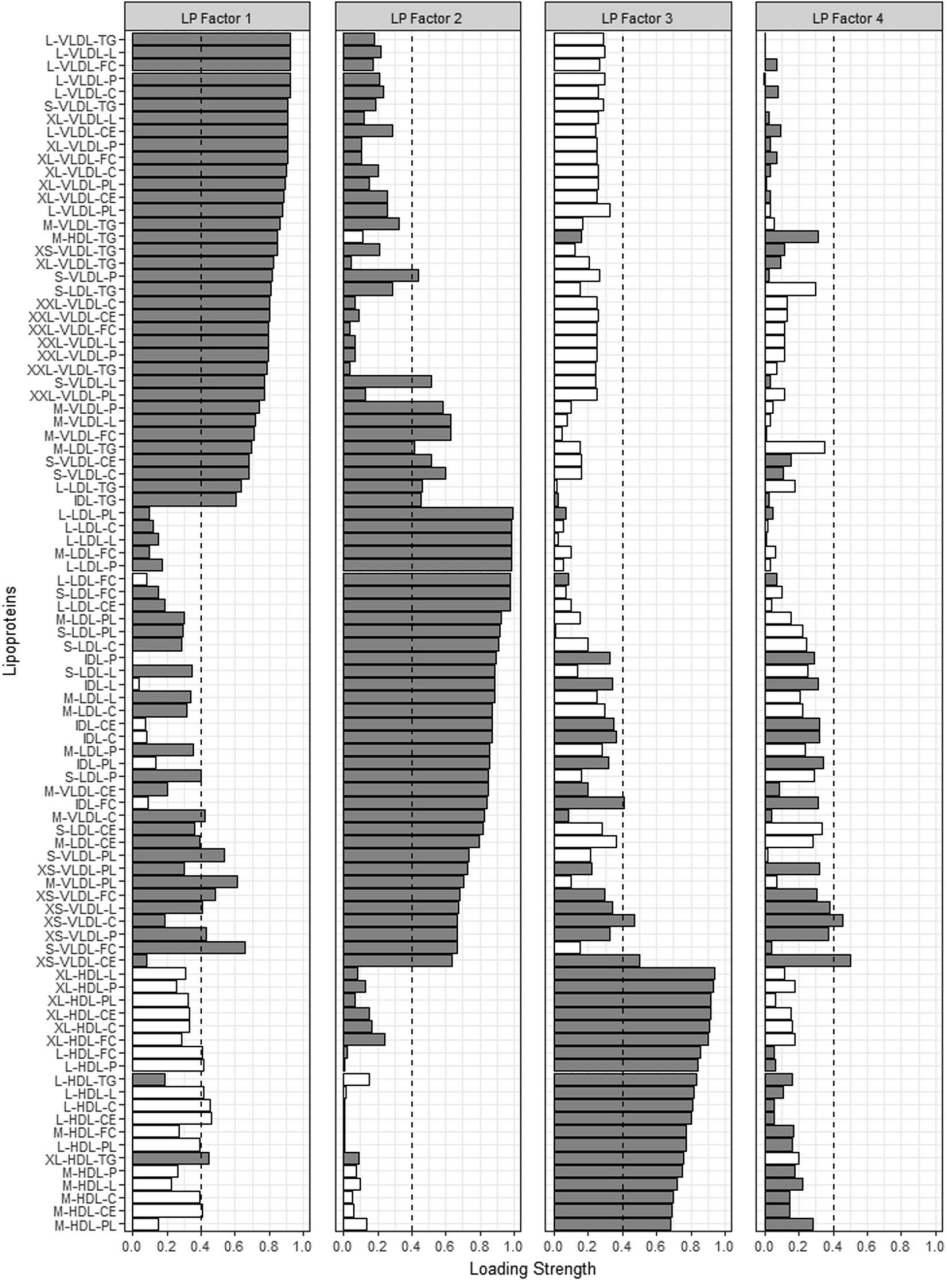
Factor loading plot for lipoprotein factors 1–4. Grey bars indicate positive loadings, white bars indicate negative loadings, dashed lines indicate the threshold of 0.4 for identifying metabolites that load on a factor. Abbreviations: C; cholesterol, CE; cholesterol ester, D; diameter, FC; free cholesterol, HDL; high density lipoprotein, IDL-intermediate density lipoprotein, L; lipids, LDL; low density lipoprotein, P; particles, PC; phosphatidylcholine, PG; phosphoglycerides, PL; phospholipid, SM; sphingomyelins, TG; triglyceride, VLDL; very low density lipoprotein.

**Figure 4:**
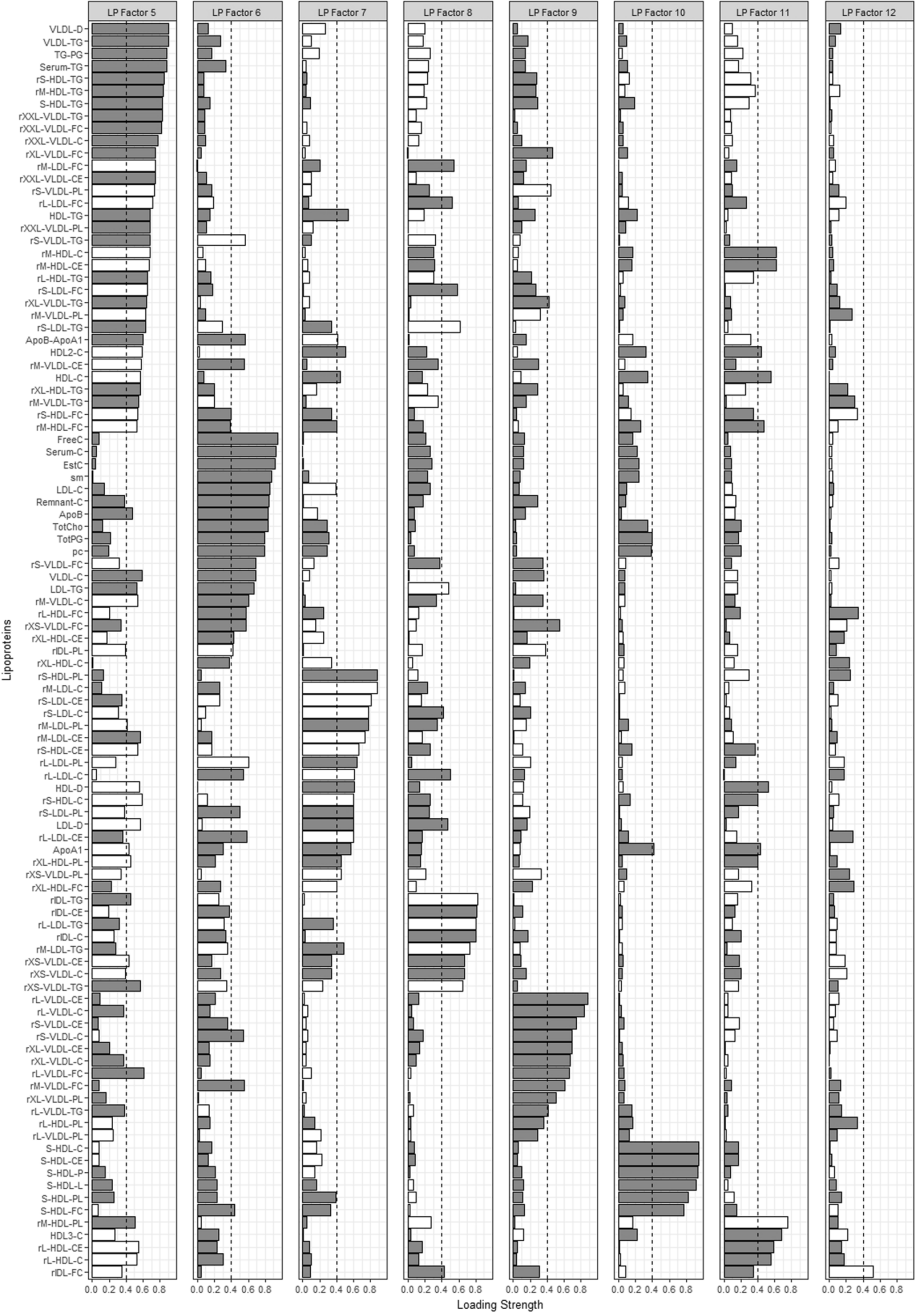
Factor loading plot for lipoprotein factors 5–12. Grey bars indicate positive loadings, white bars indicate negative loadings, dashed lines indicate the threshold of 0.4 for identifying metabolites that load on a factor. r indicates lipid ratio, e.g. rM-VLDL-C is total cholesterol to total lipids ratio in medium VLDL. Abbreviations: C; cholesterol, CE; cholesterol ester, D; diameter, FC; free cholesterol, HDL; high density lipoprotein, IDL-intermediate density lipoprotein, L; lipids, LDL; low density lipoprotein, P; particles, PC; phosphatidylcholine, PG; phosphoglycerides, PL; phospholipid, SM; sphingomyelins, TG; triglyceride, VLDL; very low density lipoprotein.

Factor analysis identified 4, 3, and 12 metabolite-containing factors for amino acids (AA), fatty acids (FA) and lipoproteins (LP), respectively. Metabolites contained in each of the factors as well as the factor loadings are shown in Figs 1–4. Associations between metabolite factors and cancer preventive behaviors that met significance thresholds are shown in Table 2. All other associations are provided in Supplementary Table 1. Similar patterns in significant metabolites were observed between the three different measures of adiposity, but there were some differences. BMI and waist circumference were both associated with six metabolite-containing factors, five of which overlapped. LP Factor 7 consisting of nine high density lipoproteins (HDL) metabolites, the ratio of apo B to A1, apo A1, nine low density lipoprotein (LDL) metabolites, and one very low density lipoprotein (VLDL) metabolite was uniquely associated with BMI, while LP Factor 8 (ten LDL, three VLDL and four intermediate density lipoprotein (IDL) metabolites) was uniquely associated with waist circumference. Body fat was associated with six metabolite-containing factors, 5 that were common with BMI or waist circumference. Not meeting recommendations for healthy body weight, was consistently associated with a decrease in factor scores ranging from −0.94 to −0.37 for FA and LP factors. The exception was LP Factor 5 which consisted of VLDL, LDL and HDL of various sizes, HDL, triglycerides (TG) and the ratio of Apo B to A1, and AA Factor 3 (glycoprotein acetylation) for which not meeting recommendations for all three body weight measures was associated with increases in factor scores.

**Table 2:** Metabolite-containing factors associated with cancer preventive behaviors that met significance thresholds.

	ß (SE)	p-value	q-value
BMI ≥ 25 kg/m^2^			
FA Factor 2	−0.69 (0.17)	5.95e-05	2.65e-4
AA Factor 3	0.69 (0.18)	<0.001	0.002
LP Factor 3	−0.94 (0.15)	1.52e-08	6.65e-07
LP Factor 5	0.42 (0.17)	0.01	0.06
LP Factor 7	−0.57 (0.17)	0.001	0.01
LP Factor 11	−0.64 (0.17)	<0.001	0.003
Waist circumference ≥102 cm (men) or ≥88 cm (women)			
FA Factor 2	−0.86 (0.16)	4.61e-07	6.16e-06
AA Factor 3	0.46 (0.18)	0.01	0.09
LP Factor 3	−0.71 (0.17)	4.69e-05	0.001
LP Factor 5	0.36 (0.17)	0.04	0.13
LP Factor 8	−0.37 (0.19)	0.05	0.15
LP Factor 11	−0.55 (0.18)	0.003	0.02
Body fat (%) ≥25% (men) or ≥30% (women)			
FA Factor 2	−0.72 (0.17)	5.27e-05	2.65e-04
AA Factor 3	0.74 (0.18)	7.73e-05	0.002
LP Factor 3	−0.65 (0.17)	<0.001	0.003
LP Factor 5	0.54 (0.17)	0.002	0.01
LP Factor 11	−0.38 (0.19)	0.05	0.14
LP Factor 12	0.41 (0.18)	0.03	0.11
Physical activity <150 min moderate or <75 min vigorous activity/d			
FA Factor 2	−0.48 (0.18)	0.01	0.03
AA Factor 1	0.36 (0.18)	0.04	0.15
AA Factor 3	0.40 (0.20)	0.04	0.15
LP Factor 3	−0.37 (0.19)	0.05	0.15
LP Factor 10	−0.42 (0.20)	0.04	0.13
LP Factor 12	0.44 (0.19)	0.02	0.09
Alcohol consumption >2 drinks/d (men) or >1 drink/d (women)			
AA Factor 1	−0.62 (0.29)	0.03	0.15
LP Factor 10	0.80 (0.32)	0.02	0.09
<5 fruits and vegetables/d			
FA Factor 3	−0.40 (0.19)	0.04	0.09

Significance determined at p < 0.05 and q < 0.20. AA: amino acids, FA: fatty acids; LP: lipoproteins.

Physical activity was associated with six metabolite-containing factors. Not meeting recommendations for physical activity was associated with lower scores for FA Factor 2, LP Factor 3 and LP Factor 10 (consisting predominately of small HDL metabolites). Not meeting recommendations was associated with higher scores for AA Factor 1 (alanine, histidine, isoleucine, leucine, valine, phenylalanine and tyrosine), AA Factor 3 and LP Factor 12 (IDL). Several factors associated with physical activity were also associated with healthy body weight measures including FA Factor 2, AA Factor 3, LP Factor 3 and LP Factor 12.

Not meeting recommendations for alcohol consumption was associated with lower scores for AA Factor 1 (ß (SE) = −0.62(0.29) and higher scores for LP Factor 10 (ß (SE) = 0.80(0.32)). Eating fewer than five fruits and vegetables was associated with lower scores for FA Factor 3 (ß (SE) = −0.40 (0.19)), which consisted of n-3 fatty acids, docosahexaenoic acid and unsaturated fatty acids.

Examination of factors across preventive behaviors identified LP Factor 7 as uniquely associated with BMI, LP Factor 8 with waist circumference and FA Factor 3 was uniquely associated with fruit and vegetable consumption. Other associations were common among at least 2 of the preventive behaviors.

## Discussion

In this analysis of 120 men and women without cancer, factor analysis of each chemical class of metabolites identified a number of associations between engagement in cancer preventive behaviors and metabolite measures. Metabolite measures associated with cancer preventive behaviors included biomarkers of inflammation, insulin sensitivity, as well as cardiometabolic risk; aberrations in lipid classes, lipid concentrations in HDL, VLDL, TG subclasses, and lipid particle size. Many of these metabolites are involved in cancer-related pathways and thus, metabolic associations may provide insight mechanisms that link engagement in cancer preventive behaviors to cancer development.

The finding of glycoprotein acetylation (the sole metabolite in AA Factor 3 that met factor loading criteria) as a metabolite positively associated with not meeting guidelines for all three measures of body weight and physical activity is notable. Glycoprotein acetylation is a recently discovered biomarker that originates from glycosylated acute-phase proteins. Our results replicate a study that found consistently lower levels of plasma glycoprotein acetylation among physically active compared to inactive individuals^14^ in a geographically and demographically distinct population; Finnish twin pairs with mean age of 28 years who were predominately male with lower mean BMI. Elevated glycoprotein acetylation has been shown to be a biomarker of insulin resistance^17^, chronic inflammation^18^, incident cardiovascular disease^19^, incident diabetes^20^, and all cause mortality^21^. We are not aware of studies that have identified relationships with cancer. The higher levels of glycoprotein acetylation associated with not meeting cancer prevention guidelines for body weight and physical activity provides insight into possible mechanistic links between preventive behaviours and cancer development, suggesting low-grade inflammation-a critical component of cancer development^22^ may underlie relationships, although causality cannot be determined with our study design. It is important to note that AA Factor 3 also contained other metabolites that did not meet our threshold for factor loading, but may have still contributed to associations with body weight and physical activity. Although, analyses between individual metabolites and continuous measures of body weight and physical activity (Supplementary Material) also found significant correlations with glycoprotein acetylation.

Not meeting recommendations for physical activity was characterized by elevated branched-chain amino acid (BCAA) metabolites: leucine, isoleucine and valine (AA Factor (3), that have previously been associated with greater BMI in a population of black older men^13^ and insulin resistance^23^. Body weight, along with physical activity and fruit and vegetable consumption were inversely associated with fatty acid factors that were characterized by fatty acids known to mediate insulin resistance; linoleic, docosahexaenoic, monounsaturated and polyunsaturated fatty acids^24^. These findings may have relevance for cancer risk as Kujala *et al*.^25^ have hypothesized that decreased catabolism of BCCAs and thus elevated levels of BCAAs in blood may be mechanistically involved in regulating fat oxidation and lead to poorer metabolic health. In addition, epidemiologic and clinical evidence suggest insulin resistance is involved in cancer development^26^.

Numerous lipids were contained within factors associated with cancer prevention guidelines for body weight, physical activity and alcohol consumption. Not meeting recommendations was generally associated with lipid profiles that are indicative of poorer metabolic health; plasma profiles were shifted towards lower HDL metabolites of varying subclasses and particle concentrations, higher ratios of VLDL and low density lipoprotein (LDL), and higher ratios of apo B to apo A1. These results replicate a prior study of four cohorts by Würtz *et al*. which differed markedly from our population of middle aged to older Canadians with respect to geography (Finland), age (mean age range among cohorts of 16–32 years), and lifestyle behaviors such as BMI (mean range 21–25 kg/m^2^) and low alcohol intake (mean range 4–5 grams/day)^15^. The study reported inverse associations between BMI and HDL (effect size −0.019 in women to −0.021 in men), and positive associations between BMI, VLDL, medium LDL and small LDL lipid metabolites^15^. The adverse metabolic profiles of physical inactivity are also consistent with a Finnish study that reported physical inactivity was characterized by higher VLDL and lower large and very large HDL metabolites, although comparison of effect sizes for metabolite-physical activity relationships between the studies is limited by differences in analytical approach as the prior studied investigated mean differences in metabolites among active and inactive individuals^14^. The results also fit within the large body of evidence linking body weight, and physical activity with decreased risk of coronary heart disease and healthier lipid profiles^27,28^.

Relative to other pathogenic pathways such as insulin resistance and inflammation, less is known about the importance of lipid metabolism and synthesis to cancer development and other aspects of cancer cell biology. Experimental evidence suggests some of the key enzymes involved in cholesterol and FA synthesis contribute to cancer cell migration and disease progression via angiogenesis, and cell invasion^29^.

Epidemiologic evidence on lipid biomarkers and cancer risk are conflicting. A meta-analysis of dyslipidemia and colorectal cancer risk suggested that high TGs and total cholesterol may be risk factors whereas low HDL may reduce the risk of colorectal cancer^30^. A meta-analysis of serum lipids and breast cancer risk only found an association between high TGs and increased breast cancer risk, and the relative risk (0.93) was modest^31^. A recent publication from the Women’s Health Study concluded that interventions for heart disease that reduce apo B or raise HDL might help to reduce cancer risk^32^.

A notable exception to the finding of generally healthier metabolic profiles among those meeting cancer prevention recommendations is alcohol consumption. Associations between AA and LP factors with alcohol consumption were in the opposite direction as those of body weight and physical activity. This may reflect the study population; participants who reported consuming more alcohol tended to be leaner and few people (9%) reported consumption above the guideline. It may also reflect that moderate alcohol consumption (i.e. above cancer prevention recommendations) is associated with cancer risk^33^ but may have a protective effect on other chronic diseases such as coronary heart disease^34^, stroke^35^ and diabetes^36^, possibly resulting in being associated with a healthier metabolic profile. In support of this notion, a previous study, which profiled alcohol consumption in nearly 10,000 young adults in Finland^16^ found complex associations between metabolites including a number of lipid metabolites with alcohol consumption that were inverse for lower consumption with subsequent convex or declining slopes. A cancer-case control study may be better suited to understand metabolite associations with alcohol consumption in the context of cancer.

A strength of our study is the availability of measured waist circumference and body fat, which are more precise indicators of excess adiposity than BMI^37^ and allowed additional exploration of measures of healthy body weight. Although it is important to note the general consistency of metabolite-containing factors associated with the three measures, which may suggest BMI is a reasonable approximation of adiposity in this population. Limitations of our study include the small sample size, which may have impeded the ability to detect many relatively strong associations and conversely, may lead to false positive associations. Although many previous metabolite associations from Finnish populations (and others) were also observed in this Canadian population, suggesting an adequate sample size, additional replication, specifically replication using similar measurement of lifestyle variables and comparable statistical approaches is needed to demonstrate the reliability of results. Measurement of metabolites in a single sample is a further limitation that does not capture past exposures related to lifestyle factors or daily variability in metabolites or past exposures. A previous study has shown modest variability in metabolite levels over time^38^. Participants were not required to provide fasting blood samples as part of the cohort protocol and we cannot rule out the possible effect of variable fasting status on metabolite levels, particularly lipids. The finding of a sole association between metabolite-factors with fruit and vegetable consumption may reflect the metabolite platform used, which is dominated by fatty acids and has been mainly developed to capture metabolites associated with cardiometabolic risk. As the recommendations for cancer prevention are similar to those of other disease prevention guidelines including cardiovascular disease, the use of the platform for our study objectives is appropriate, however, metabolites previously correlated with dietary intake of fruits and vegetables did not overlap with the metabolites measured here^39,40^. It is also possible that associations were masked due to the limitations of self-reported cancer preventive behaviors that are prone to bias. However, a number of factors associated with self-reported physical activity and alcohol consumption overlapped with factors associated with measured adiposity, which increases our confidence in the findings. The use of factor analysis may have also influenced the observation of metabolite-lifestyle relationships as factors are identified that explain the most variance in metabolites which are not necessarily factors that are the most predictive for lifestyle behaviors or cancer prevention.

In conclusion, our study shows cancer preventive behaviors are associated with complex metabolic signatures. The results confirm prior findings and also provide new insight into the diverse molecular processes related to adiposity, physical activity, alcohol and fruit and vegetable consumption. Biomarkers of cancer preventive behaviors could serve as mechanistic links and may help to further our understanding of the complex relationships between preventive behaviors and cancer development. Larger studies are needed to validate findings and genetic data is needed to assess causality of metabolite-lifestyle relationships and cancer risk. Prospective cancer case control studies are also warranted to establish a direct link between metabolites and cancer.

## Methods

### Population

The BC Generations Project (BCGP), British Columbia’s (BC’s) largest-ever cancer prevention study and part of the national Canadian Partnership for Tomorrow Project^41^ is a prospective, longitudinal cohort study. Between 2009 and 2014, approximately 30,000 participants aged 35–69 from across BC were enrolled in the study and will be followed for up to 50 years. Participants completed standard lifestyle and health questionnaires, provided blood samples and approximately 90% were either assessed for physical measurements or provided self-measured physical measurements. For this pilot study, all participants who met the following criteria were identified: no history of smoking or cancer at baseline, not pregnant, were assessed for physical measures, provided blood samples within 90 days of completing the questionnaire and physical assessments and had a blood processing time (collection to storage) of less than 24 hours. From this population, a random sample of 60 men and 60 women were chosen. The sample size of 120 reflects the exploratory nature of our research aim.

### Lifestyle variable*s*

Fruits and vegetables consumed in a typical day were self-reported as the number of servings of fresh, frozen, canned or cooked fruits and vegetables. The average number, amount and type of alcoholic drinks in a week were self-reported. A drink was defined as 12 ounces of beer, 5 ounces of wine or 1.5 ounces of 80-proof liquor/spirits. Trained personnel assessed adiposity measures. Height was measured using a stadiometer. Weight and percent body fat were measured with bioelectrical impedance (Tanita BC-418, Tanita Corporation of America Inc.). Waist circumference was measured to the nearest inch using a tape measure aligned to the top of the iliac crest. Time spent physically active and sedentary in the prior week was assessed by the short-form International Physical Activity Questionnaire (IPAQ-SF). IPAQ-SF is a validated questionnaire that captures different domains of physical activity (at work, at home, active transportation and leisure time) including time spent sitting and physical effort of physical activity (light, moderate and vigorous)^42^. Five participants were missing information on physical activity, all other variables were complete for the 120 participants.

### Engagement in cancer prevention behavior

Cancer prevention guidelines from the WCRF/AICR^4^ were used to define engagement in cancer prevention. We focused on the four main guidelines for cancer prevention; (1) achieve and maintain a healthy weight throughout life, (2) be physically active, (3) eat a healthy diet with an emphasis on plant foods, and (4) limit alcohol intake. Engagement in preventive behavior was defined as yes/no. Waist circumference and body fat were additionally investigated as measures of healthy body weight as they are more precise indicators of excess adiposity than BMI^37^. Cut points of waist circumference <102 cm in men and <88 cm in women^43^ and body fat <25% in men and <30% in women^44^ were selected from previous definitions of normal weight and overweight/obesity. Translation of preventive guidelines to the BCGP population is shown in Table 3.

**Table 3:** Behavioral cancer prevention recommendations and translation to prevention score in the BCGP.

Recommendation	Does not meet recommendation	Meets recommendation
Achieve and maintain a healthy weight throughout life	BMI ≥25 kg/m^2^ or <18.5 kg/m^2^	BMI 18.5–24.9 kg/m^2^
	Waist circumference ≥102 cm (men) or ≥88 cm (women)	Waist circumference <102 cm (men) or <88 cm (women)
	Body fat ≥25% (men) or ≥30% (women)	Body fat <25% (men) or <30% (women)
At least 150 min of moderate intensity or 75 min of vigorous intensity physical activity per week	<150 min of moderate or <75 min vigorous physical activity per week	≥150 min of moderate or ≥75 min vigorous physical activity per week
Eat at least 2 1/2 cups of vegetables and fruit each day	<5 servings	≥5 servings
Drink no more than 1 drink per day for women or 2 per day for men	>1 drink/d women, >2 drinks/d men	≤1 drink/d women, ≤2 drinks/d men

### Blood collection

Non-fasting blood was drawn at a commercial community medical laboratory following standard blood collection techniques^45^. Fasting status has minimal effect on most metabolites^38^. After collection, blood was temporarily stored at 4 °C onsite of the medical laboratory and then shipped on ice to the processing laboratory for arrival the following day. Blood was processed as per BCGP’s Standard Operating Procedure^46^. Briefly, blood collected in EDTA vacutainer tubes were centrifuged for 10 minutes at 1300 g/4 °C/with brake on to separate out the plasma fraction. Plasma was transferred to a cryovial and stored at −80 °C within 24 hours post blood collection. Samples were visually noted to be free of hemolysis and lipidemia.

### Metabolite profiling

Plasma metabolite profiling was performed by Nightingale Health Ltd. (Vantaa, Finland). The platform has been applied in a number of epidemiologic studies^12,21,47^, and the detailed protocol has been previously published^48^. The high-throughput, fully automated NMR platform provided quantification of lipoprotein lipids and subclasses, fatty acids (FAs) and FA composition, ketone bodies, amino acids, glycolysis and gluconeogenesis-related metabolites. A full list of metabolite measures is provided in Supplementary Table 2. Collectively, 225 metabolite measures were captured by the platform. Of these, two metabolites; glucose and lactate levels were excluded for a total of 223 metabolites as glucose levels were noted to be very low while lactate levels were substantially higher than commonly observed, indicating glycolysis. Otherwise, the laboratory report indicated high sample quality: metabolite distributions matched those observed in other large cohort studies, all samples passed quality control measures that check for sample irregularities and signs of degradation. The average success rate of biomarker quantification was 98.7%.

### Statistical Analysis

Metabolite measures were log-transformed and standardized to approximate a normal distribution with mean 0 and variance 1. Missing metabolites were assumed to be below the limit of detection and were imputed with half of the minimum value of the non-missing metabolites^13^ as only lipid levels in the VLDL were below the detection limit, which was expected as concentrations are biologically close to zero. Factor analysis was performed to reduce the multiple metabolites into a smaller set of latent variables. Metabolites that are highly correlated with one another formed a common factor. Factor analysis was performed separately for each major chemical class. Factor groups included one containing 15 metabolites (eight amino acids, ketone bodies; acetoacetate, 3-hydroxybutyrate and miscellaneous metabolites citrate, acetate, creatinine, albumin and glycoprotein acetylation) referred to as AA, FA containing 16 metabolites and two LP-containing groups. LPs were divided into two groups due to the sample size (N = 120 participants), one containing extra-large to medium VLDL particle concentrations, intermediate density lipoproteins, low density lipoproteins and HDL of all sizes (91 metabolites; LP Factors 1–4), and the other containing primarily ratios of particle concentrations to total lipids (101 metabolites, LP Factors 5–12). An orthogonal varimax rotation was used. Parallel analysis and scree plots were used to select the number of factors retained (Supplementary Fig. 1). Individual metabolites with a component loading of 0.4 or greater were considered as being contained within each factor.

Multivariable linear regression was used to evaluate the association between individual cancer preventive behaviors and metabolite-containing factors. Cancer preventive behaviors were scored as 0 for meeting the guideline or 1 for not meeting. Models were adjusted for age, gender, and education. To account for multiple testing, false discovery rates were computed with use of the q value method for each exposure separately^49^. Statistical significance was set at p < 0.05 and q < 0.20 to account for multiple comparisons. Significance levels in prior exploratory analyses^50–53^ guided the choice of statistical significance, which we propose is reasonable in a small pilot study. Although the study aims and thus analyses were focused on metabolic associations with respect to engagement in cancer preventive lifestyle recommendations, associations between individual metabolites and continuous measures of BMI, physical activity, alcohol consumption and fruit and vegetable consumption were determined (see Supplementary Tables 3–6) to facilitate comparison with prior and future studies. Linear regression adjusted for age, gender and education was used to estimate change in lifestyle behavior per 1-standard deviation increase in log-transformed metabolite.

Statistical analyses were conducted using R version 3.3.1 (R Foundation for Statistical Computing, Vienna, Austria) and Stata version 14.0 (Stata Inc., TX, USA). The study protocol was approved by the joint Research Ethics Board of the University of British Columbia and British Columbia Cancer Agency (Approval Number: H17-00004). All research was performed in accordance with guidelines and regulations. All study participants provided written informed consent.

## Electronic supplementary material

Supplementary Material

## Data Availability

The datasets generated during and/or analyzed during the current study are available from the corresponding author on reasonable request and with approval from the BC Generations Project.
