# Supplementary material for: Metabolic profiling of adherence to diet, physical activity and body size recommendations for cancer prevention
Source: Sci Rep. 2018 Nov 2;8:16293. doi: 10.1038/s41598-018-34662-7 (PMC6214951; doi:10.1038/s41598-018-34662-7)
Supplement: Supplementary file 1 — Supplementary Material [file 41598_2018_34662_MOESM1_ESM.docx]

**Metabolic profiling of adherence to diet, physical activity and body size recommendations for cancer prevention**

Qianqian Gu^1^, John J Spinelli^1,2^, Trevor BJ Dummer^1^, Treena E McDonald^2^, Steven C Moore^3^, and Rachel A Murphy^1*^

^1^School of Population and Public Health, University of British Columbia, Vancouver, BC, ^2^Cancer Control Research, BC Cancer Agency, Vancouver, BC, ^3^Division of Cancer Epidemiology & Genetics, National Cancer Institute, Bethesda, MD

**Supplementary Table 1.** Metabolite-containing factors and cancer preventive behaviors, associations that did not meet significance thresholds

|  | ß (SE) | p-value | q-value |
| --- | --- | --- | --- |
| BMI ≥25 kg/m^2^ |  |  |  |
| FA Factor 1 | 0.04 (0.19) | 0.84 | 0.66 |
| FA Factor 3 | 0.11 (0.18) | 0.54 | 0.56 |
| AA Factor 1 | 0.22 (0.17) | 0.20 | 0.49 |
| AA Factor 2 | 0.09 (0.16) | 0.59 | 0.75 |
| AA Factor 4 | 0.12 (0.16) | 0.45 | 0.75 |
| LP Factor 1 | 0.26 (0.17) | 0.14 | 0.20 |
| LP Factor 2 | 0.03 (0.19) | 0.86 | 0.58 |
| LP Factor 4 | -0.13 (0.19) | 0.48 | 0.45 |
| LP Factor 6 | -0.08 (0.19) | 0.68 | 0.52 |
| LP Factor 8 | -0.26 (0.19) | 0.17 | 0.22 |
| LP Factor 9 | -0.2 (0.19) | 0.30 | 0.31 |
| LP Factor 10 | 0.04 (0.19) | 0.85 | 0.58 |
| LP Factor 12 | 0.29 (0.18) | 0.10 | 0.17 |
| Waist circumference ≥102cm (men) or ≥88 cm (women) | | | |
| FA Factor 1 | -0.19 (0.19) | 0.33 | 0.50 |
| FA Factor 3 | -0.32 (0.18) | 0.08 | 0.18 |
| AA Factor 1 | 0.20 (0.17) | 0.24 | 0.54 |
| AA Factor 2 | -0.06 (0.16) | 0.71 | 0.75 |
| AA Factor 4 | -0.05 (0.16) | 0.75 | 0.75 |
| LP Factor 1 | 0.21 (0.18) | 0.10 | 0.17 |
| LP Factor 2 | -0.25 (0.19) | 0.20 | 0.23 |
| LP Factor 4 | -0.05 (0.19) | 0.80 | 0.56 |
| LP Factor 6 | -0.29 (0.19) | 0.13 | 0.19 |
| LP Factor 7 | -0.25 (0.18) | 0.17 | 0.22 |
| LP Factor 9 | -0.25 (0.19) | 0.19 | 0.23 |
| LP Factor 10 | 0.09 (0.19) | 0.64 | 0.52 |
| LP Factor 12 | 0.18 (0.18) | 0.33 | 0.32 |
| Body fat (%) ≥25% (men) or ≥30% (women) | | |  |
| FA Factor 1 | 0.15 (0.20) | 0.43 | 0.53 |
| FA Factor 3 | -0.15 (0.19) | 0.45 | 0.53 |
| AA Factor 1 | 0.03 (0.18) | 0.85 | 0.81 |
| AA Factor 2 | 0.10 (0.16) | 0.55 | 0.75 |
| AA Factor 4 | -0.15 (0.17) | 0.35 | 0.71 |
| LP Factor 1 | 0.34 (0.18) | 0.07 | 0.15 |
| LP Factor 2 | 0.01 (0.20) | 0.97 | 0.60 |
| LP Factor 4 | 0.02 (0.19) | 0.94 | 0.59 |
| LP Factor 6 | -0.11 (0.20) | 0.57 | 0.48 |
| LP Factor 7 | -0.29 (0.17) | 0.10 | 0.16 |
| LP Factor 8 | 0.02 (0.19) | 0.91 | 0.59 |
| LP Factor 9 | -0.33 (0.19) | 0.09 | 0.16 |
| LP Factor 10 | 0.33 (0.20) | 0.10 | 0.16 |
| Physical activity <150min moderate or <75min vigorous activity/d | | |  |
| FA Factor 1 | -0.09 (0.20) | 0.65 | 0.60 |
| FA Factor 3 | 0.04 (0.19) | 0.83 | 0.66 |
| AA Factor 2 | 0.00 (0.17) | 0.98 | 0.82 |
| AA Factor 4 | 0.09 (0.17) | 0.61 | 0.75 |
| FA Factor 1 | -0.09 (0.20) | 0.65 | 0.60 |
| FA Factor 3 | 0.04 (0.19) | 0.84 | 0.66 |
| LP Factor 1 | 0.27 (0.19) | 0.15 | 0.20 |
| LP Factor 2 | -0.13 (0.2) | 0.52 | 0.45 |
| LP Factor 4 | 0.06 (0.20) | 0.76 | 0.56 |
| LP Factor 5 | 0.30 (0.18) | 0.09 | 0.16 |
| LP Factor 6 | -0.16 (0.20) | 0.44 | 0.42 |
| LP Factor 7 | -0.23 (0.19) | 0.24 | 0.27 |
| LP Factor 8 | -0.06 (0.20) | 0.76 | 0.56 |
| LP Factor 9 | 0.22 (0.20) | 0.28 | 0.29 |
| LP Factor 11 | -0.09 (0.19) | 0.65 | 0.52 |
| <5 fruits and vegetables/d |  |  |  |
| FA Factor 1 | 0.02 (0.20) | 0.93 | 0.69 |
| FA Factor 2 | -0.18 (0.18) | 0.34 | 0.50 |
| AA Factor 1 | -0.02 (0.18) | 0.92 | 0.81 |
| AA Factor 2 | -0.05 (0.17) | 0.75 | 0.75 |
| AA Factor 3 | 0.02 (0.19) | 0.93 | 0.81 |
| AA Factor 4 | 0.11 (0.17) | 0.52 | 0.75 |
| LP Factor 1 | 0.04 (0.18) | 0.81 | 0.56 |
| LP Factor 2 | 0.13 (0.20) | 0.50 | 0.45 |
| LP Factor 3 | -0.08 (0.18) | 0.68 | 0.52 |
| LP Factor 4 | 0.28 (0.19) | 0.15 | 0.20 |
| LP Factor 5 | -0.02 (0.18) | 0.93 | 0.59 |
| LP Factor 6 | 0.02 (0.20) | 0.91 | 0.59 |
| LP Factor 7 | 0.07 (0.19) | 0.70 | 0.53 |
| LP Factor 8 | 0.00 (0.19) | 0.99 | 0.60 |
| LP Factor 9 | 0.10 (0.20) | 0.63 | 0.52 |
| LP Factor 10 | 0.30 (0.20) | 0.13 | 0.19 |
| LP Factor 11 | -0.32 (0.19) | 0.09 | 0.16 |
| LP Factor 12 | 0.12 (0.19) | 0.51 | 0.45 |
| Alcohol consumption >2 drinks/d (men) or >1 drink/d (women) | | | |
| FA Factor 1 | -0.23 (0.33) | 0.48 | 0.53 |
| FA Factor 2 | 0.13 (0.31) | 0.68 | 0.60 |
| FA Factor 3 | 0.46 (0.31) | 0.15 | 0.29 |
| AA Factor 2 | -0.11 (0.28) | 0.70 | 0.75 |
| AA Factor 3 | -0.51 (0.32) | 0.11 | 0.33 |
| AA Factor 4 | -0.12 (0.28) | 0.66 | 0.75 |
| LP Factor 1 | -0.29 (0.30) | 0.35 | 0.34 |
| LP Factor 2 | 0.01 (0.33) | 0.97 | 0.60 |
| LP Factor 3 | 0.57 (0.30) | 0.06 | 0.15 |
| LP Factor 4 | 0.59 (0.32) | 0.07 | 0.15 |
| LP Factor 5 | -0.49 (0.29) | 0.09 | 0.16 |
| LP Factor 6 | -0.09 (0.33) | 0.78 | 0.56 |
| LP Factor 7 | 0.58 (0.30) | 0.06 | 0.15 |
| LP Factor 8 | 0.41 (0.32) | 0.20 | 0.23 |
| LP Factor 9 | 0.33 (0.32) | 0.31 | 0.32 |
| LP Factor 11 | -0.21 (0.31) | 0.50 | 0.45 |
| LP Factor 12 | -0.49 (0.31) | 0.12 | 0.18 |

Significance determined at p<0.05 and q<0.20.

**Supplementary Table 2.** Metabolic measures in the sample of 120 participants from the BCGP

| Metabolic measures | Description |
| --- | --- |
| XXL-VLDL-P | Concentration of chylomicrons and extremely large VLDL particles |
| XXL-VLDL-L | Total lipids in chylomicrons and extremely large VLDL |
| XXL-VLDL-PL | Phospholipids in chylomicrons and extremely large VLDL |
| XXL-VLDL-C | Total cholesterol in chylomicrons and extremely large VLDL |
| XXL-VLDL-CE | Cholesterol esters in chylomicrons and extremely large VLDL |
| XXL-VLDL-FC | Free cholesterol in chylomicrons and extremely large VLDL |
| XXL-VLDL-TG | Triglycerides in chylomicrons and extremely large VLDL |
| XL-VLDL-P | Concentration of very large VLDL particles |
| XL-VLDL-L | Total lipids in very large VLDL |
| XL-VLDL-PL | Phospholipids in very large VLDL |
| XL-VLDL-C | Total cholesterol in very large VLDL |
| XL-VLDL-CE | Cholesterol esters in very large VLDL |
| XL-VLDL-FC | Free cholesterol in very large VLDL |
| XL-VLDL-TG | Triglycerides in very large VLDL |
| L-VLDL-P | Concentration of large VLDL particles |
| L-VLDL-L | Total lipids in large VLDL |
| L-VLDL-PL | Phospholipids in large VLDL |
| L-VLDL-C | Total cholesterol in large VLDL |
| L-VLDL-CE | Cholesterol esters in large VLDL |
| L-VLDL-FC | Free cholesterol in large VLDL |
| L-VLDL-TG | Triglycerides in large VLDL |
| M-VLDL-P | Concentration of medium VLDL particles |
| M-VLDL-L | Total lipids in medium VLDL |
| M-VLDL-PL | Phospholipids in medium VLDL |
| M-VLDL-C | Total cholesterol in medium VLDL |
| M-VLDL-CE | Cholesterol esters in medium VLDL |
| M-VLDL-FC | Free cholesterol in medium VLDL |
| M-VLDL-TG | Triglycerides in medium VLDL |
| S-VLDL-P | Concentration of small VLDL particles |
| S-VLDL-L | Total lipids in small VLDL |
| S-VLDL-PL | Phospholipids in small VLDL |
| S-VLDL-C | Total cholesterol in small VLDL |
| S-VLDL-CE | Cholesterol esters in small VLDL |
| S-VLDL-FC | Free cholesterol in small VLDL |
| S-VLDL-TG | Triglycerides in small VLDL |
| XS-VLDL-P | Concentration of very small VLDL particles |
| XS-VLDL-L | Total lipids in very small VLDL |
| XS-VLDL-PL | Phospholipids in very small VLDL |
| XS-VLDL-C | Total cholesterol in very small VLDL |
| XS-VLDL-CE | Cholesterol esters in very small VLDL |
| XS-VLDL-FC | Free cholesterol in very small VLDL |
| XS-VLDL-TG | Triglycerides in very small VLDL |
| IDL-P | Concentration of IDL particles |
| IDL-L | Total lipids in IDL |
| IDL-PL | Phospholipids in IDL |
| IDL-C | Total cholesterol in IDL |
| IDL-CE | Cholesterol esters in IDL |
| IDL-FC | Free cholesterol in IDL |
| IDL-TG | Triglycerides in IDL |
| L-LDL-P | Concentration of large LDL particles |
| L-LDL-L | Total lipids in large LDL |
| L-LDL-PL | Phospholipids in large LDL |
| L-LDL-C | Total cholesterol in large LDL |
| L-LDL-CE | Cholesterol esters in large LDL |
| L-LDL-FC | Free cholesterol in large LDL |
| L-LDL-TG | Triglycerides in large LDL |
| M-LDL-P | Concentration of medium LDL particles |
| M-LDL-L | Total lipids in medium LDL |
| M-LDL-PL | Phospholipids in medium LDL |
| M-LDL-C | Total cholesterol in medium LDL |
| M-LDL-CE | Cholesterol esters in medium LDL |
| M-LDL-FC | Free cholesterol in medium LDL |
| M-LDL-TG | Triglycerides in medium LDL |
| S-LDL-P | Concentration of small LDL particles |
| S-LDL-L | Total lipids in small LDL |
| S-LDL-PL | Phospholipids in small LDL |
| S-LDL-C | Total cholesterol in small LDL |
| S-LDL-CE | Cholesterol esters in small LDL |
| S-LDL-FC | Free cholesterol in small LDL |
| S-LDL-TG | Triglycerides in small LDL |
| XL-HDL-P | Concentration of very large HDL particles |
| XL-HDL-L | Total lipids in very large HDL |
| XL-HDL-PL | Phospholipids in very large HDL |
| XL-HDL-C | Total cholesterol in very large HDL |
| XL-HDL-CE | Cholesterol esters in very large HDL |
| XL-HDL-FC | Free cholesterol in very large HDL |
| XL-HDL-TG | Triglycerides in very large HDL |
| L-HDL-P | Concentration of large HDL particles |
| L-HDL-L | Total lipids in large HDL |
| L-HDL-PL | Phospholipids in large HDL |
| L-HDL-C | Total cholesterol in large HDL |
| L-HDL-CE | Cholesterol esters in large HDL |
| L-HDL-FC | Free cholesterol in large LDL |
| L-HDL-TG | Triglycerides in large HDL |
| M-HDL-P | Concentration of medium HDL particles |
| M-HDL-L | Total lipids in medium HDL |
| M-HDL-PL | Phospholipids in medium HDL |
| M-HDL-C | Total cholesterol in medium HDL |
| M-HDL-CE | Cholesterol esters in medium HDL |
| M-HDL-FC | Free cholesterol in medium HDL |
| M-HDL-TG | Triglycerides in medium HDL |
| S-HDL-P | Concentration of small HDL particles |
| S-HDL-L | Total lipids in small HDL |
| S-HDL-PL | Phospholipids in small HDL |
| S-HDL-C | Total cholesterol in small HDL |
| S-HDL-CE | Cholesterol esters in small HDL |
| S-HDL-FC | Free cholesterol in small HDL |
| S-HDL-TG | Triglycerides in small HDL |
| XXL-VLDL-PL_% | Phospholipids to total lipids ratio in chylomicrons and extremely large VLDL |
| XXL-VLDL-C_% | Total cholesterol to total lipids ratio in chylomicrons and extremely large VLDL |
| XXL-VLDL-CE_% | Cholesterol esters to total lipids ratio in chylomicrons and extremely large VLDL |
| XXL-VLDL-FC_% | Free cholesterol to total lipids ratio in chylomicrons and extremely large VLDL |
| XXL-VLDL-TG_% | Triglycerides to total lipids ratio in chylomicrons and extremely large VLDL |
| XL-VLDL-PL_% | Phospholipids to total lipids ratio in very large VLDL |
| XL-VLDL-C_% | Total cholesterol to total lipids ratio in very large VLDL |
| XL-VLDL-CE_% | Cholesterol esters to total lipids ratio in very large VLDL |
| XL-VLDL-FC_% | Free cholesterol to total lipids ratio in very large VLDL |
| XL-VLDL-TG_% | Triglycerides to total lipids ratio in large VLDL |
| L-VLDL-PL_% | Phospholipids to total lipids ratio in large VLDL |
| L-VLDL-C_% | Total cholesterol to total lipids ratio in large VLDL |
| L-VLDL-CE_% | Cholesterol esters to total lipids ratio in large VLDL |
| L-VLDL-FC_% | Free cholesterol to total lipids ratio in large VLDL |
| L-VLDL-TG_% | Triglycerides to total lipids ratio in large VLDL |
| M-VLDL-PL_% | Phospholipids to total lipids ratio in medium VLDL |
| M-VLDL-C_% | Total cholesterol to total lipids ratio in medium VLDL |
| M-VLDL-CE_% | Cholesterol esters to total lipids ratio in medium VLDL |
| M-VLDL-FC_% | Free cholesterol to total lipids ratio in medium VLDL |
| M-VLDL-TG_% | Triglycerides to total lipids ratio in medium VLDL |
| S-VLDL-PL_% | Phospholipids to total lipids ratio in small VLDL |
| S-VLDL-C_% | Total cholesterol to total lipids ratio in small VLDL |
| S-VLDL-CE_% | Cholesterol esters to total lipids ratio in small VLDL |
| S-VLDL-FC_% | Free cholesterol to total lipids ratio in small VLDL |
| S-VLDL-TG_% | Triglycerides to total lipids ratio in small VLDL |
| XS-VLDL-PL_% | Phospholipids to total lipids ratio in very small VLDL |
| XS-VLDL-C_% | Total cholesterol to total lipids in very small VLDL |
| XS-VLDL-CE_% | Cholesterol esters to total lipids ratio in very small VLDL |
| XS-VLDL-FC_% | Free cholesterol to total lipids ratio in very small VLDL |
| XS-VLDL-TG_% | Triglycerides to total lipids ratio in very small VLDL |
| IDL-PL_% | Phospholipids to total lipids ratio in IDL |
| IDL-C_% | Total cholesterol to total lipids ratio in IDL |
| IDL-CE_% | Cholesterol esters to total lipids ratio in IDL |
| IDL-FC_% | Free cholesterol to total lipids ratio in IDL |
| IDL-TG_% | Triglycerides to total lipids ratio in IDL |
| L-LDL-PL_% | Phospholipids to total lipids ratio in large LDL |
| L-LDL-C_% | Total cholesterol to total lipids ratio in large LDL |
| L-LDL-CE_% | Cholesterol esters to total lipids ratio in large LDL |
| L-LDL-FC_% | Free cholesterol to total lipids ratio in large LDL |
| L-LDL-TG_% | Triglycerides to total lipids ratio in large LDL |
| M-LDL-PL_% | Phospholipids to total lipids ratio in medium LDL |
| M-LDL-C_% | Total cholesterol to total lipids ratio in medium LDL |
| M-LDL-CE_% | Cholesterol esters to total lipids ratio in medium LDL |
| M-LDL-FC_% | Free cholesterol to total lipids ratio in medium LDL |
| M-LDL-TG_% | Triglycerides to total lipids ratio in medium LDL |
| S-LDL-PL_% | Phospholipids to total lipids ratio in small LDL |
| S-LDL-C_% | Phospholipids to total lipids ratio in small LDL |
| S-LDL-CE_% | Cholesterol esters to total lipids ratio in small LDL |
| S-LDL-FC_% | Free cholesterol to total lipids ratio in small LDL |
| S-LDL-TG_% | Triglycerides to total lipids ratio in small LDL |
| XL-HDL-PL_% | Phospholipids to total lipids ratio in very large LDL |
| XL-HDL-C_% | Total cholesterol to total lipids ratio in very large HDL |
| XL-HDL-CE_% | Cholesterol esters to total lipids ratio in very large HDL |
| XL-HDL-FC_% | Free cholesterol to total lipids ratio in very large HDL |
| XL-HDL-TG_% | Triglycerides to total lipids ratio in very large HDL |
| L-HDL-PL_% | Phospholipids to total lipids ratio in large HDL |
| L-HDL-C_% | Total cholesterol to lipids ratio in large HDL |
| L-HDL-CE_% | Cholesterol esters to total lipids ratio in large HDL |
| L-HDL-FC_% | Free cholesterol to total lipids ratio in large HDL |
| L-HDL-TG_% | Triglycerides to total lipids ratio in large HDL |
| M-HDL-PL_% | Phospholipids to total lipids ratio in medium HDL |
| M-HDL-C_% | Total cholesterol to total lipids ratio in medium HDL |
| M-HDL-CE_% | Cholesterol esters to total lipids ratio in medium HDL |
| M-HDL-FC_% | Free cholesterol to total lipids ratio in medium HDL |
| M-HDL-TG_% | Triglycerides to total lipids ratio in medium HDL |
| S-HDL-PL_% | Phospholipids to total lipids ratio in small HDL |
| S-HDL-C_% | Total cholesterol to total lipids ratio in small HDL |
| S-HDL-CE_% | Cholesterol esters to total lipids ratio in small HDL |
| S-HDL-FC_% | Free cholesterol to total lipids ratio in small HDL |
| S-HDL-TG_% | Triglycerides to total lipids ratio in small HDL |
| VLDL-D | Mean diameter for VLDL particles |
| LDL-D | Mean diameter for LDL particles |
| HDL-D | Mean diameter for HDL particles |
| Serum-C | Serum total cholesterol |
| VLDL-C | Total cholesterol in VLDL |
| Remnant-C | Remnant cholesterol (non-HDL, non-LDL cholesterol) |
| LDL-C | Total cholesterol in LDL |
| HDL-C | Total cholesterol in HDL |
| HDL2-C | Total cholesterol in HDL2 |
| HDL3-C | Total cholesterol in HDL3 |
| EstC | Esterified cholesterol |
| FreeC | Free cholesterol |
| Serum-TG | Serum total triglycerides |
| VLDL-TG | Triglycerides in VLDL |
| LDL-TG | Triglycerides in LDL |
| HDL-TG | Triglycerides in HDL |
| TotPG | Total phosphoglycerides |
| TG/PG | Ratio of triglycerides to phosphoglycerides |
| PC | Phosphatidylcholine and other cholines |
| SM | Sphingomyelins |
| TotCho | Total cholines |
| ApoA1 | Apolipoprotein A-1 |
| ApoB | Apolipoprotein B |
| ApoB/ApoA1 | Ratio of apolipoprotein B to apolipoprotein A-1 |
| TotFA | Total fatty acids |
| UnSat | Estimated degree of unsaturation |
| DHA | 22:6, docosahexaenoic acid |
| LA | 18:2, linoleic acid |
| FAn3 | Omega-3 fatty acids |
| FAn6 | Omega-6 fatty acids |
| PUFA | Polyunsaturated fatty acids |
| MUFA | Monounsaturated fatty acids; 16:1, 18:1 |
| SFA | Saturated fatty acids |
| DHA/FA | Ratio of 22:6 docosahexaenoic acid to total fatty acids |
| LA/FA | Ratio of 18:2 linoleic acid to total fatty acids |
| FAn3/FA | Ratio of omega-3 fatty acids to total fatty acids |
| FAn6/FA | Ratio of omega-6 fatty acids to total fatty acids |
| PUFA/FA | Ratio of polyunsaturated fatty acids to total fatty acids |
| MUFA/FA | Ratio of monounsaturated fatty acids to total fatty acids |
| SFA/FA | Ratio of saturated fatty acids to total fatty acids |
| Cit | Citrate |
| Ala | Alanine |
| Gln | Glutamine |
| His | Histidine |
| Ile | Isoleucine |
| Leu | Leucine |
| Val | Valine |
| Phe | Phenylalanine |
| Tyr | Tyrosine |
| Ace | Acetate |
| AcAce | Acetoacetate |
| bOHBut | 3-hydroxybutyrate |
| Crea | Creatinine |
| Alb | Albumin |
| Gp | Glycoprotein acetylation |

**Supplementary Table 3.** Metabolites associated with BMI on a continuous scale

|  | ß (SE) | p-value | q-value |
| --- | --- | --- | --- |
| XXL-VLDL-P | 0.07 (0.18) | 0.57 | 0.24 |
| XXL-VLDL-L | 0.06 (0.19) | 0.62 | 0.25 |
| XXL-VLDL-PL | 0.06 (0.16) | 0.64 | 0.25 |
| XXL-VLDL-C | 0.05 (0.20) | 0.73 | 0.27 |
| XXL-VLDL-CE | 0.05 (0.18) | 0.68 | 0.26 |
| XXL-VLDL-FC | 0.03 (0.24) | 0.83 | 0.30 |
| XXL-VLDL-TG | 0.04 (0.24) | 0.73 | 0.27 |
| XL-VLDL-P | 0.34 (0.13) | 2.5e-04 | 3.33e-04 |
| XL-VLDL-L | 0.34 (0.13) | 2.2e-04 | 2.99e-04 |
| XL-VLDL-PL | 0.36 (0.11) | 1.0e-04 | 1.58e-04 |
| XL-VLDL-C | 0.32 (0.13) | 6.2e-04 | 7.36e-04 |
| XL-VLDL-CE | 0.31 (0.12) | 1.2e-03 | 0.001 |
| XL-VLDL-FC | 0.33 (0.19) | 4.1e-04 | 5.10e-04 |
| XL-VLDL-TG | 0.30 (0.19) | 1.3e-03 | 0.001 |
| L-VLDL-P | 0.34 (0.10) | 1.0e-04 | 1.58e-04 |
| L-VLDL-L | 0.34 (0.11) | 1.0e-04 | 1.58e-04 |
| L-VLDL-PL | 0.36 (0.10) | 4.2e-05 | 8.19e-05 |
| L-VLDL-C | 0.32 (0.12) | 3.0e-04 | 3.80e-04 |
| L-VLDL-CE | 0.30 (0.12) | 6.7e-04 | 7.83e-04 |
| L-VLDL-FC | 0.33 (0.15) | 1.5e-04 | 2.11e-04 |
| L-VLDL-TG | 0.33 (0.11) | 1.1e-04 | 1.67e-04 |
| M-VLDL-P | 0.21 (0.07) | 0.02 | 0.15 |
| M-VLDL-L | 0.19 (0.07) | 0.03 | 0.02 |
| M-VLDL-PL | 0.21 (0.06) | 0.02 | 0.02 |
| M-VLDL-C | 0.05 (0.09) | 0.63 | 0.25 |
| M-VLDL-CE | -0.02 (0.11) | 0.80 | 0.29 |
| M-VLDL-FC | 0.15 (0.08) | 0.09 | 0.05 |
| M-VLDL-TG | 0.28 (0.08) | 1.1e-03 | 0.001 |
| S-VLDL-P | 0.23 (0.06) | 8.6e-03 | 0.007 |
| S-VLDL-L | 0.21 (0.06) | 0.02 | 0.02 |
| S-VLDL-PL | 0.20 (0.05) | 0.03 | 0.02 |
| S-VLDL-C | 0.12 (0.08) | 0.19 | 0.10 |
| S-VLDL-CE | 0.09 (0.09) | 0.32 | 0.15 |
| S-VLDL-FC | 0.14 (0.07) | 0.12 | 0.07 |
| S-VLDL-TG | 0.27 (0.06) | 0.001 | 0.001 |
| XS-VLDL-P | -0.08 (0.04) | 0.40 | 0.18 |
| XS-VLDL-L | -0.09 (0.04) | 0.37 | 0.17 |
| XS-VLDL-PL | -0.02 (0.04) | 0.85 | 0.30 |
| XS-VLDL-C | -0.20 (0.05) | 0.04 | 0.03 |
| XS-VLDL-CE | -0.23 (0.06) | 0.01 | 0.008 |
| XS-VLDL-FC | -0.06 (0.05) | 0.52 | 0.22 |
| XS-VLDL-TG | 0.21 (0.04) | 0.02 | 0.02 |
| IDL-P | -0.16 (0.04) | 0.10 | 0.06 |
| IDL-L | -0.17 (0.04) | 0.08 | 0.05 |
| IDL-PL | -0.15 (0.04) | 0.11 | 0.06 |
| IDL-C | -0.20 (0.04) | 0.04 | 0.03 |
| IDL-CE | -0.19 (0.05) | 0.04 | 0.03 |
| IDL-FC | -0.22 (0.04) | 0.02 | 0.02 |
| IDL-TG | 0.13 (0.03) | 0.17 | 0.09 |
| L-LDL-P | 0.06 (0.04) | 0.53 | 0.22 |
| L-LDL-L | 0.04 (0.04) | 0.64 | 0.25 |
| L-LDL-PL | -0.0 (0.04) | 0.98 | 0.34 |
| L-LDL-C | 0.05 (0.04) | 0.60 | 0.24 |
| L-LDL-CE | 0.08 (0.05) | 0.37 | 0.17 |
| L-LDL-FC | -0.06 (0.04) | 0.53 | 0.22 |
| L-LDL-TG | 0.16 (0.03) | 0.09 | 0.05 |
| M-LDL-P | 0.20 (0.04) | 0.03 | 0.02 |
| M-LDL-L | 0.19 (0.04) | 0.04 | 0.03 |
| M-LDL-PL | 0.14 (0.04) | 0.13 | 0.07 |
| M-LDL-C | 0.20 (0.05) | 0.03 | 0.02 |
| M-LDL-CE | 0.25 (0.05) | 0.006 | 0.005 |
| M-LDL-FC | 0.06 (0.04) | 0.51 | 0.22 |
| M-LDL-TG | 0.24 (0.04) | 0.007 | 0.006 |
| S-LDL-P | 0.16 (0.04) | 0.08 | 0.05 |
| S-LDL-L | 0.14 (0.04) | 0.14 | 0.08 |
| S-LDL-PL | 0.08 (0.04) | 0.41 | 0.18 |
| S-LDL-C | 0.15 (0.04) | 0.11 | 0.06 |
| S-LDL-CE | 0.21 (0.04) | 0.02 | 0.02 |
| S-LDL-FC | 0.06 (0.04) | 0.52 | 0.22 |
| S-LDL-TG | 0.24 (0.04) | 6.8e-03 | 0.006 |
| XL-HDL-P | -0.50 (0.06) | 2.9e-09 | 2.45e-08 |
| XL-HDL-L | -0.49 (0.08) | 1.3e-09 | 2.45e-08 |
| XL-HDL-PL | -0.47 (0.13) | 7.7e-09 | 4.88e-08 |
| XL-HDL-C | -0.48 (0.07) | 6.1e-09 | 4.21e-08 |
| XL-HDL-CE | -0.48 (0.08) | 5.1e-09 | 3.88e-08 |
| XL-HDL-FC | -0.47 (0.04) | 2.6e-08 | 1.41e-07 |
| XL-HDL-TG | -0.30 (0.05) | 0.001 | 0.001 |
| L-HDL-P | -0.49 (0.09) | 4.2e-10 | 1.82e-10 |
| L-HDL-L | -0.46 (0.09) | 2.2e-09 | 2.45e-08 |
| L-HDL-PL | -0.44 (0.09) | 2.8e-08 | 1.42e-07 |
| L-HDL-C | -0.47 (0.10) | 2.3e-09 | 2.45e-08 |
| L-HDL-CE | -0.46 (0.11) | 2.8e-09 | 2.45e-08 |
| L-HDL-FC | -0.47 (0.09) | 1.5e-09 | 2.45e-08 |
| L-HDL-TG | -0.46 (0.05) | 7.8e-08 | 3.49e-07 |
| M-HDL-P | -0.37 (0.03) | 2.3e-06 | 6.99e-06 |
| M-HDL-L | -0.33 (0.03) | 2.3e-05 | 5.14e-05 |
| M-HDL-PL | -0.28 (0.03) | 4.5e-04 | 5.43e-04 |
| M-HDL-C | -0.37 (0.04) | 2.0e-06 | 6.61e-06 |
| M-HDL-CE | -0.37 (0.04) | 2.3e-06 | 6.99e-06 |
| M-HDL-FC | -0.37 (0.04) | 2.0e-06 | 6.61e-06 |
| M-HDL-TG | 0.05 (0.05) | 0.57 | 0.24 |
| S-HDL-P | 0.03 (0.02) | 0.79 | 0.29 |
| S-HDL-L | 0.04 (0.02) | 0.72 | 0.27 |
| S-HDL-PL | 0.02 (0.02) | 0.85 | 0.30 |
| S-HDL-C | -0.03 (0.02) | 0.73 | 0.27 |
| S-HDL-CE | -0.01 (0.02) | 0.88 | 0.31 |
| S-HDL-FC | -0.17 (0.02) | 0.06 | 0.04 |
| S-HDL-TG | 0.24 (0.05) | 0.005 | 0.005 |
| XXL-VLDL-PL_% | -0.03 (0.07) | 0.79 | 0.29 |
| XXL-VLDL-C_% | -0.07 (0.05) | 0.58 | 0.24 |
| XXL-VLDL-CE_% | -0.01 (0.05) | 0.93 | 0.32 |
| XXL-VLDL-FC_% | -0.08 (0.07) | 0.52 | 0.22 |
| XXL-VLDL-TG_% | -0.02 (0.09) | 0.88 | 0.31 |
| XL-VLDL-PL_% | -0.15 (0.04) | 0.12 | 0.07 |
| XL-VLDL-C_% | -0.12 (0.04) | 0.23 | 0.11 |
| XL-VLDL-CE_% | -0.22 (0.05) | 0.03 | 0.02 |
| XL-VLDL-FC_% | 0.24 (0.07) | 0.01 | 0.008 |
| XL-VLDL-TG_% | 0.14 (0.10) | 0.17 | 0.09 |
| L-VLDL-PL_% | -0.10 (0.03) | 0.31 | 0.14 |
| L-VLDL-C_% | 0.09 (0.03) | 0.35 | 0.16 |
| L-VLDL-CE_% | -0.07 (0.04) | 0.46 | 0.20 |
| L-VLDL-FC_% | 0.25 (0.05) | 0.005 | 0.005 |
| L-VLDL-TG_% | 0.05 (0.01) | 0.60 | 0.24 |
| M-VLDL-PL_% | -0.07 (0.02) | 0.44 | 0.19 |
| M-VLDL-C_% | -0.23 (0.04) | 0.01 | 0.008 |
| M-VLDL-CE_% | -0.22 (0.08) | 0.02 | 0.02 |
| M-VLDL-FC_% | -0.06 (0.02) | 0.55 | 0.23 |
| M-VLDL-TG_% | 0.28 (0.04) | 0.001 | 0.001 |
| S-VLDL-PL_% | -0.11 (0.22) | 0.20 | 0.10 |
| S-VLDL-C_% | -0.15 (0.03) | 0.12 | 0.07 |
| S-VLDL-CE_% | -0.11 (0.04) | 0.24 | 0.12 |
| S-VLDL-FC_% | -0.19 (0.02) | 0.04 | 0.03 |
| S-VLDL-TG_% | 0.21 (0.03) | 0.02 | 0.02 |
| XS-VLDL-PL_% | 0.23 (0.01) | 0.01 | 0.008 |
| XS-VLDL-C_% | -0.36 (0.02) | 9.3e-05 | 1.57e-04 |
| XS-VLDL-CE_% | -0.35 (0.03) | 1.3e-04 | 1.94e-04 |
| XS-VLDL-FC_% | 0.05 (0.01) | 0.61 | 0.24 |
| XS-VLDL-TG_% | 0.33 (0.04) | 1.5e-04 | 2.11e-04 |
| IDL-PL_% | 0.12 (0.01) | 0.20 | 0.10 |
| IDL-C_% | -0.30 (0.01) | 0.001 | 0.001 |
| IDL-CE_% | -0.25 (0.01) | 0.008 | 0.007 |
| IDL-FC_% | -0.34 (0.01) | 3.0e-04 | 3.80e-04 |
| IDL-TG_% | 0.28 (0.04) | 0.003 | 0.003 |
| L-LDL-PL_% | -0.25 (0.01) | 0.005 | 0.005 |
| L-LDL-C_% | 0.08 (0.01) | 0.38 | 0.17 |
| L-LDL-CE_% | 0.27 (0.01) | 0.003 | 0.003 |
| L-LDL-FC_% | -0.34 (0.01) | 8.5e-05 | 1.47e-04 |
| L-LDL-TG_% | 0.10 (0.03) | 0.29 | 0.14 |
| M-LDL-PL_% | -0.35 (0.01) | 3.8e-05 | 7.6e-05 |
| M-LDL-C_% | 0.23 (0.01) | 0.01 | 0.01 |
| M-LDL-CE_% | 0.38 (0.01) | 2.9e-06 | 8.48e-06 |
| M-LDL-FC_% | -0.36 (0.01) | 2.2e-05 | 5.07e-05 |
| M-LDL-TG_% | 0.03 (0.03) | 0.75 | 0.28 |
| S-LDL-PL_% | -0.33 (0.01) | 1.4e-04 | 2.05e-04 |
| S-LDL-C_% | 0.11 (0.01) | 0.24 | 0.12 |
| S-LDL-CE_% | 0.34 (0.01) | 3.5e-05 | 7.19e-05 |
| S-LDL-FC_% | -0.23 (0.01) | 0.01 | 0.008 |
| S-LDL-TG_% | 0.15 (0.04) | 0.10 | 0.06 |
| XL-HDL-PL_% | -0.38 (0.05) | 3.5e-06 | 7.19e-05 |
| XL-HDL-C_% | 0.45 (0.02) | 6.6e-08 | 3.14e-07 |
| XL-HDL-CE_% | 0.30 (0.02) | 9.2e-04 | 0.001 |
| XL-HDL-FC_% | 0.49 (0.04) | 1.9e-09 | 2.45e-08 |
| XL-HDL-TG_% | 0.38 (0.08) | 3.7e-06 | 1.00e-05 |
| L-HDL-PL_% | 0.10 (0.02) | 0.28 | 0.13 |
| L-HDL-C_% | -0.37 (0.20) | 1.9e-05 | 4.51e-05 |
| L-HDL-CE_% | -0.37 (0.02) | 1.7e-05 | 4.17e-05 |
| L-HDL-FC_% | -0.14 (0.02) | 0.13 | 0.07 |
| L-HDL-TG_% | 0.27 (0.08) | 9.3e-04 | 0.001 |
| M-HDL-PL_% | 0.41 (0.01) | 1.8e-06 | 6.51e-06 |
| M-HDL-C_% | -0.36 (0.02) | 2.9e-05 | 6.30e-05 |
| M-HDL-CE_% | -0.34 (0.02) | 7.0e-05 | 1.27e-04 |
| M-HDL-FC_% | -0.42 (0.01) | 3.0e-07 | 1.20e-06 |
| M-HDL-TG_% | 0.25 (0.06) | 0.003 | 0.003 |
| S-HDL-PL_% | -0.05 (0.01) | 0.61 | 0.24 |
| S-HDL-C_% | -0.13 (0.01) | 0.18 | 0.10 |
| S-HDL-CE_% | -0.08 (0.01) | 0.39 | 0.17 |
| S-HDL-FC_% | -0.38 (0.01) | 8.2e-06 | 2.15e-05 |
| S-HDL-TG_% | 0.26 (0.04) | 0.002 | 0.002 |
| VLDL-D | 0.33 (0.01) | 8.2e-05 | 1.45e-04 |
| LDL-D | -0.37 (0.00) | 1.1e-05 | 2.79e-05 |
| HDL-D | -0.48 (0.00) | 4.8e-10 | 1.82e-8 |
| Serum-C | -0.11 (0.03) | 0.27 | 0.13 |
| VLDL-C | 0.13 (0.08) | 0.15 | 0.08 |
| Remnant-C | 0.04 (0.06) | 0.69 | 0.27 |
| LDL-C | 0.10 (0.04) | 0.27 | 0.13 |
| HDL-C | -0.44 (0.04) | 2.1e-08 | 1.23e-07 |
| HDL2-C | -0.41 (0.05) | 2.9e-07 | 1.20 |
| HDL3-C | -0.34 (0.03) | 1.6e-04 | 2.2e-04 |
| EstC | -0.11 (0.03) | 0.25 | 0.12 |
| FreeC | -0.09 (0.03) | 0.35 | 0.16 |
| Serum-TG | 0.27 (0.07) | 0.002 | 0.002 |
| VLDL-TG | 0.28 (0.10) | 7.4e-04 | 8.5e-04 |
| LDL-TG | 0.20 (0.03) | 0.03 | 0.02 |
| HDL-TG | -0.04 (0.04) | 0.64 | 0.25 |
| TotPG | -0.14 (0.03) | 0.15 | 0.08 |
| TG/PG | 0.34 (0.06) | 3.3e-05 | 6.97e-05 |
| PC | -0.16 (0.03) | 0.09 | 0.05 |
| SM | -0.12 (0.03) | 0.22 | 0.11 |
| TotCho | -0.17 (0.03) | 0.07 | 0.05 |
| ApoA1 | -0.41 (0.02) | 3.6e-07 | 1.37e-06 |
| ApoB | 0.12 (0.03) | 0.18 | 0.10 |
| ApoB/ApoA1 | 0.30 (0.04) | 4.4e-04 | 5.39e-04 |
| TotFA | 0.11 (0.04) | 0.23 | 0.11 |
| UnSat | -0.18 (0.01) | 0.04 | 0.03 |
| DHA | 0.07 (0.06) | 0.44 | 0.19 |
| LA | -0.03 (0.04) | 0.72 | 0.27 |
| FAn3 | 0.11 (0.05) | 0.25 | 0.12 |
| FAn6 | -0.02 (0.04) | 0.88 | 0.31 |
| PUFA | 0.01 (0.04) | 0.90 | 0.31 |
| MUFA | 0.19 (0.05) | 0.04 | 0.03 |
| SFA | 0.11 (0.04) | 0.22 | 0.11 |
| DHA/FA | -0.00 (0.05) | 0.98 | 0.34 |
| LA/FA | -0.34 (0.02) | 1.1e-04 | 1.67e-04 |
| FAn3/FA | 0.04 (0.03) | 0.71 | 0.27 |
| FAn6/FA | -0.35 (0.01) | 4.4e-05 | 8.36e-05 |
| PUFA/FA | -0.29 (0.01) | 8.0e-04 | 9.08e-04 |
| MUFA/FA | 0.32 (0.01) | 2.7e-04 | 3.54e-4 |
| SFA/FA | 0.01 (0.01) | 0.92 | 0.32 |
| Cit | -0.16 (0.03) | 0.09 | 0.05 |
| Ala | 0.12 (0.03) | 0.22 | 0.11 |
| Gln | -0.05 (0.03) | 0.58 | 0.24 |
| His | -0.02 (0.03) | 0.83 | 0.30 |
| Ile | 0.17 (0.06) | 0.04 | 0.03 |
| Leu | 0.14 (0.05) | 0.08 | 0.05 |
| Val | 0.14 (0.05) | 0.10 | 0.06 |
| Phe | 0.11 (0.02) | 0.26 | 0.13 |
| Tyr | 0.02 (0.04) | 0.82 | 0.30 |
| Ace | 0.00 (0.05) | 0.99 | 0.34 |
| AcAce | 0.11 (0.08) | 0.22 | 0.11 |
| bOHBut | 0.03 (0.06) | 0.75 | 0.28 |
| Crea | 0.11 (0.03) | 0.11 | 0.06 |
| Alb | -0.05 (0.01) | 0.63 | 0.25 |
| Gp | 0.37 (0.03) | 4.6e-05 | 8.53e-05 |

Significance determined at p<0.05 and q<0.20. Beta-regression coefficients (standard errors) indicate change in BMI in kg/m^2^ per 1-standard deviation increase in log-transformed metabolite adjusted for age, gender and education.

**Supplementary Table 4.** Metabolites associated with physical activity on a continuous scale

|  | ß (SE) | p-value | q-value |
| --- | --- | --- | --- |
| XXL-VLDL-P |  |  |  |
| XXL-VLDL-L |  |  |  |
| XXL-VLDL-PL |  |  |  |
| XXL-VLDL-C |  |  |  |
| XXL-VLDL-CE |  |  |  |
| XXL-VLDL-FC |  |  |  |
| XXL-VLDL-TG |  |  |  |
| XL-VLDL-P |  |  |  |
| XL-VLDL-L |  |  |  |
| XL-VLDL-PL |  |  |  |
| XL-VLDL-C |  |  |  |
| XL-VLDL-CE |  |  |  |
| XL-VLDL-FC |  |  |  |
| XL-VLDL-TG |  |  |  |
| L-VLDL-P |  |  |  |
| L-VLDL-L |  |  |  |
| L-VLDL-PL |  |  |  |
| L-VLDL-C |  |  |  |
| L-VLDL-CE |  |  |  |
| L-VLDL-FC |  |  |  |
| L-VLDL-TG |  |  |  |
| M-VLDL-P |  |  |  |
| M-VLDL-L |  |  |  |
| M-VLDL-PL |  |  |  |
| M-VLDL-C |  |  |  |
| M-VLDL-CE |  |  |  |
| M-VLDL-FC |  |  |  |
| M-VLDL-TG |  |  |  |
| S-VLDL-P |  |  |  |
| S-VLDL-L |  |  |  |
| S-VLDL-PL |  |  |  |
| S-VLDL-C |  |  |  |
| S-VLDL-CE |  |  |  |
| S-VLDL-FC |  |  |  |
| S-VLDL-TG |  |  |  |
| XS-VLDL-P |  |  |  |
| XS-VLDL-L |  |  |  |
| XS-VLDL-PL |  |  |  |
| XS-VLDL-C |  |  |  |
| XS-VLDL-CE |  |  |  |
| XS-VLDL-FC |  |  |  |
| XS-VLDL-TG |  |  |  |
| IDL-P |  |  |  |
| IDL-L |  |  |  |
| IDL-PL |  |  |  |
| IDL-C |  |  |  |
| IDL-CE |  |  |  |
| IDL-FC |  |  |  |
| IDL-TG |  |  |  |
| L-LDL-P |  |  |  |
| L-LDL-L |  |  |  |
| L-LDL-PL |  |  |  |
| L-LDL-C |  |  |  |
| L-LDL-CE |  |  |  |
| L-LDL-FC |  |  |  |
| L-LDL-TG |  |  |  |
| M-LDL-P |  |  |  |
| M-LDL-L |  |  |  |
| M-LDL-PL |  |  |  |
| M-LDL-C |  |  |  |
| M-LDL-CE |  |  |  |
| M-LDL-FC |  |  |  |
| M-LDL-TG |  |  |  |
| S-LDL-P |  |  |  |
| S-LDL-L |  |  |  |
| S-LDL-PL |  |  |  |
| S-LDL-C |  |  |  |
| S-LDL-CE |  |  |  |
| S-LDL-FC |  |  |  |
| S-LDL-TG |  |  |  |
| XL-HDL-P |  |  |  |
| XL-HDL-L |  |  |  |
| XL-HDL-PL |  |  |  |
| XL-HDL-C |  |  |  |
| XL-HDL-CE |  |  |  |
| XL-HDL-FC |  |  |  |
| XL-HDL-TG |  |  |  |
| L-HDL-P |  |  |  |
| L-HDL-L |  |  |  |
| L-HDL-PL |  |  |  |
| L-HDL-C |  |  |  |
| L-HDL-CE |  |  |  |
| L-HDL-FC |  |  |  |
| L-HDL-TG |  |  |  |
| M-HDL-P |  |  |  |
| M-HDL-L |  |  |  |
| M-HDL-PL |  |  |  |
| M-HDL-C |  |  |  |
| M-HDL-CE |  |  |  |
| M-HDL-FC |  |  |  |
| M-HDL-TG |  |  |  |
| S-HDL-P |  |  |  |
| S-HDL-L |  |  |  |
| S-HDL-PL |  |  |  |
| S-HDL-C |  |  |  |
| S-HDL-CE |  |  |  |
| S-HDL-FC |  |  |  |
| S-HDL-TG |  |  |  |
| XXL-VLDL-PL_% |  |  |  |
| XXL-VLDL-C_% |  |  |  |
| XXL-VLDL-CE_% |  |  |  |
| XXL-VLDL-FC_% |  |  |  |
| XXL-VLDL-TG_% |  |  |  |
| XL-VLDL-PL_% |  |  |  |
| XL-VLDL-C_% |  |  |  |
| XL-VLDL-CE_% |  |  |  |
| XL-VLDL-FC_% |  |  |  |
| XL-VLDL-TG_% |  |  |  |
| L-VLDL-PL_% |  |  |  |
| L-VLDL-C_% |  |  |  |
| L-VLDL-CE_% |  |  |  |
| L-VLDL-FC_% |  |  |  |
| L-VLDL-TG_% |  |  |  |
| M-VLDL-PL_% |  |  |  |
| M-VLDL-C_% |  |  |  |
| M-VLDL-CE_% |  |  |  |
| M-VLDL-FC_% |  |  |  |
| M-VLDL-TG_% |  |  |  |
| S-VLDL-PL_% |  |  |  |
| S-VLDL-C_% |  |  |  |
| S-VLDL-CE_% |  |  |  |
| S-VLDL-FC_% |  |  |  |
| S-VLDL-TG_% |  |  |  |
| XS-VLDL-PL_% |  |  |  |
| XS-VLDL-C_% |  |  |  |
| XS-VLDL-CE_% |  |  |  |
| XS-VLDL-FC_% |  |  |  |
| XS-VLDL-TG_% |  |  |  |
| IDL-PL_% |  |  |  |
| IDL-C_% |  |  |  |
| IDL-CE_% |  |  |  |
| IDL-FC_% |  |  |  |
| IDL-TG_% |  |  |  |
| L-LDL-PL_% |  |  |  |
| L-LDL-C_% |  |  |  |
| L-LDL-CE_% |  |  |  |
| L-LDL-FC_% |  |  |  |
| L-LDL-TG_% |  |  |  |
| M-LDL-PL_% |  |  |  |
| M-LDL-C_% |  |  |  |
| M-LDL-CE_% |  |  |  |
| M-LDL-FC_% |  |  |  |
| M-LDL-TG_% |  |  |  |
| S-LDL-PL_% |  |  |  |
| S-LDL-C_% |  |  |  |
| S-LDL-CE_% |  |  |  |
| S-LDL-FC_% |  |  |  |
| S-LDL-TG_% |  |  |  |
| XL-HDL-PL_% |  |  |  |
| XL-HDL-C_% |  |  |  |
| XL-HDL-CE_% |  |  |  |
| XL-HDL-FC_% |  |  |  |
| XL-HDL-TG_% |  |  |  |
| L-HDL-PL_% |  |  |  |
| L-HDL-C_% |  |  |  |
| L-HDL-CE_% |  |  |  |
| L-HDL-FC_% |  |  |  |
| L-HDL-TG_% |  |  |  |
| M-HDL-PL_% |  |  |  |
| M-HDL-C_% |  |  |  |
| M-HDL-CE_% |  |  |  |
| M-HDL-FC_% |  |  |  |
| M-HDL-TG_% |  |  |  |
| S-HDL-PL_% |  |  |  |
| S-HDL-C_% |  |  |  |
| S-HDL-CE_% |  |  |  |
| S-HDL-FC_% |  |  |  |
| S-HDL-TG_% |  |  |  |
| VLDL-D |  |  |  |
| LDL-D |  |  |  |
| HDL-D |  |  |  |
| Serum-C |  |  |  |
| VLDL-C |  |  |  |
| Remnant-C |  |  |  |
| LDL-C |  |  |  |
| HDL-C |  |  |  |
| HDL2-C |  |  |  |
| HDL3-C |  |  |  |
| EstC |  |  |  |
| FreeC |  |  |  |
| Serum-TG |  |  |  |
| VLDL-TG |  |  |  |
| LDL-TG |  |  |  |
| HDL-TG |  |  |  |
| TotPG |  |  |  |
| TG/PG |  |  |  |
| PC |  |  |  |
| SM |  |  |  |
| TotCho |  |  |  |
| ApoA1 |  |  |  |
| ApoB |  |  |  |
| ApoB/ApoA1 |  |  |  |
| TotFA |  |  |  |
| UnSat |  |  |  |
| DHA |  |  |  |
| LA |  |  |  |
| FAn3 |  |  |  |
| FAn6 |  |  |  |
| PUFA |  |  |  |
| MUFA |  |  |  |
| SFA |  |  |  |
| DHA/FA |  |  |  |
| LA/FA |  |  |  |
| FAn3/FA |  |  |  |
| FAn6/FA |  |  |  |
| PUFA/FA |  |  |  |
| MUFA/FA |  |  |  |
| SFA/FA |  |  |  |
| Cit |  |  |  |
| Ala |  |  |  |
| Gln |  |  |  |
| His |  |  |  |
| Ile |  |  |  |
| Leu |  |  |  |
| Val |  |  |  |
| Phe |  |  |  |
| Tyr |  |  |  |
| Ace |  |  |  |
| AcAce |  |  |  |
| bOHBut |  |  |  |
| Crea |  |  |  |
| Alb |  |  |  |
| Gp |  |  |  |

Significance determined at p<0.05 and q<0.20. Beta-regression coefficients (standard errors) indicate change in minutes of physical activity per 1-standard deviation increase in log-transformed metabolite adjusted for age, gender and education.

**Supplementary Table 5.** Metabolites associated with fruits and vegetables on a continuous scale

|  | ß (SE) | p-value | q-value |
| --- | --- | --- | --- |
| XXL-VLDL-P | 0.05 (0.04) | 0.72 |  |
| XXL-VLDL-L | 0.05 (0.04) | 0.72 |  |
| XXL-VLDL-PL | 0.04 (0.03) | 0.76 |  |
| XXL-VLDL-C | 0.07 (0.04) | 0.59 |  |
| XXL-VLDL-CE | 0.08 (0.03) | 0.53 |  |
| XXL-VLDL-FC | 0.05 (0.05) | 0.69 |  |
| XXL-VLDL-TG | 0.05 (0.05) | 0.69 |  |
| XL-VLDL-P | 0.00 (0.03) | 0.98 |  |
| XL-VLDL-L | -0.00 (0.03) | 0.98 |  |
| XL-VLDL-PL | 0.02 (0.03) | 0.88 |  |
| XL-VLDL-C | 0.03 (0.03) | 0.79 |  |
| XL-VLDL-CE | 0.04 (0.03) | 0.66 |  |
| XL-VLDL-FC | -0.02 (0.04) | 0.81 |  |
| XL-VLDL-TG | 0.01 (0.04) | 0.90 |  |
| L-VLDL-P | -0.05 (0.02) | 0.61 |  |
| L-VLDL-L | -0.05 (0.02) | 0.60 |  |
| L-VLDL-PL | -0.03 (0.02) | 0.76 |  |
| L-VLDL-C | -0.04 (0.03) | 0.69 |  |
| L-VLDL-CE | -0.02 (0.03) | 0.80 |  |
| L-VLDL-FC | -0.04 (0.03) | 0.63 |  |
| L-VLDL-TG | -0.06 (0.02) | 0.51 |  |
| M-VLDL-P | -0.09 (0.02) | 0.33 |  |
| M-VLDL-L | -0.09 (0.02) | 0.33 |  |
| M-VLDL-PL | -0.07 (0.01) | 0.47 |  |
| M-VLDL-C | -0.05 (0.02) | 0.59 |  |
| M-VLDL-CE | -0.01 (0.02) | 0.92 |  |
| M-VLDL-FC | -0.10 (0.02) | 0.30 |  |
| M-VLDL-TG | -0.11 (0.02) | 0.24 |  |
| S-VLDL-P | -0.08 (0.01) | 0.36 |  |
| S-VLDL-L | -0.08 (0.01) | 0.37 |  |
| S-VLDL-PL | -0.04 (0.01) | 0.67 |  |
| S-VLDL-C | -0.09 (0.02) | 0.31 |  |
| S-VLDL-CE | -0.11 (0.02) | 0.23 |  |
| S-VLDL-FC | -0.07 (0.01) | 0.43 |  |
| S-VLDL-TG | -0.08 (0.01) | 0.34 |  |
| XS-VLDL-P | -0.01 (0.01) | 0.95 |  |
| XS-VLDL-L | -0.00 (0.01) | 0.99 |  |
| XS-VLDL-PL | 0.01 (0.01) | 0.90 |  |
| XS-VLDL-C | 0.04 (0.01) | 0.70 |  |
| XS-VLDL-CE | 0.05 (0.01) | 0.60 |  |
| XS-VLDL-FC | -0.00 (0.01) | 0.99 |  |
| XS-VLDL-TG | -0.10 (0.01) | 0.26 |  |
| IDL-P | -0.07 (0.01) | 0.49 |  |
| IDL-L | -0.06 (0.01) | 0.51 |  |
| IDL-PL | -0.05 (0.01) | 0.60 |  |
| IDL-C | -0.07 (0.01) | 0.50 |  |
| IDL-CE | -0.08 (0.01) | 0.44 |  |
| IDL-FC | -0.04 (0.01) | 0.72 |  |
| IDL-TG | -0.07 (0.01) | 0.49 |  |
| L-LDL-P | -0.09 (0.01) | 0.38 |  |
| L-LDL-L | -0.08 (0.01) | 0.39 |  |
| L-LDL-PL | -0.08 (0.01) | 0.42 |  |
| L-LDL-C | -0.08 (0.01) | 0.39 |  |
| L-LDL-CE | -0.09 (0.01) | 0.36 |  |
| L-LDL-FC | -0.06 (0.01) | 0.52 |  |
| L-LDL-TG | -0.07 (0.01) | 0.48 |  |
| M-LDL-P | -0.08 (0.01) | 0.41 |  |
| M-LDL-L | -0.08 (0.01) | 0.39 |  |
| M-LDL-PL | -0.08 (0.01) | 0.42 |  |
| M-LDL-C | -0.08 (0.01) | 0.38 |  |
| M-LDL-CE | -0.08 (0.01) | 0.41 |  |
| M-LDL-FC | -0.09 (0.01) | 0.37 |  |
| M-LDL-TG | -0.04 (0.01) | 0.63 |  |
| S-LDL-P | -0.05 (0.01) | 0.57 |  |
| S-LDL-L | -0.06 (0.01) | 0.53 |  |
| S-LDL-PL | -0.04 (0.01) | 0.66 |  |
| S-LDL-C | -0.07 (0.01) | 0.47 |  |
| S-LDL-CE | -0.05 (0.01) | 0.57 |  |
| S-LDL-FC | -0.09 (0.01) | 0.37 |  |
| S-LDL-TG | -0.04 (0.01) | 0.66 |  |
| XL-HDL-P | 0.17 (0.02) | 0.07 |  |
| XL-HDL-L | 0.17 (0.02) | 0.06 |  |
| XL-HDL-PL | 0.14 (0.03) | 0.13 |  |
| XL-HDL-C | 0.18 (0.02) | 0.05 |  |
| XL-HDL-CE | 0.18 (0.02) | 0.04 |  |
| XL-HDL-FC | 0.14 (0.01) | 0.12 |  |
| XL-HDL-TG | 0.08 (0.01) | 0.40 |  |
| L-HDL-P | 0.09 (0.02) | 0.32 |  |
| L-HDL-L | 0.08 (0.02) | 0.36 |  |
| L-HDL-PL | 0.07 (0.02) | 0.42 |  |
| L-HDL-C | 0.07 (0.03) | 0.40 |  |
| L-HDL-CE | 0.07 (0.03) | 0.41 |  |
| L-HDL-FC | 0.08 (0.02) | 0.33 |  |
| L-HDL-TG | 0.11 (0.01) | 0.26 |  |
| M-HDL-P | 0.05 (0.01) | 0.57 |  |
| M-HDL-L | 0.03 (0.01) | 0.69 |  |
| M-HDL-PL | 0.01 (0.01) | 0.88 |  |
| M-HDL-C | 0.04 (0.01) | 0.64 |  |
| M-HDL-CE | 0.04 (0.01) | 0.67 |  |
| M-HDL-FC | 0.07 (0.01) | 0.41 |  |
| M-HDL-TG | -0.02 (0.01) | 0.81 |  |
| S-HDL-P | -0.13 (0.00) | 0.18 |  |
| S-HDL-L | -0.13 (0.00) | 0.17 |  |
| S-HDL-PL | -0.13 (0.00) | 0.17 |  |
| S-HDL-C | -0.12 (0.00) | 0.23 |  |
| S-HDL-CE | -0.13 (0.00) |  |  |
| S-HDL-FC |  |  |  |
| S-HDL-TG |  |  |  |
| XXL-VLDL-PL_% |  |  |  |
| XXL-VLDL-C_% |  |  |  |
| XXL-VLDL-CE_% |  |  |  |
| XXL-VLDL-FC_% |  |  |  |
| XXL-VLDL-TG_% |  |  |  |
| XL-VLDL-PL_% |  |  |  |
| XL-VLDL-C_% |  |  |  |
| XL-VLDL-CE_% |  |  |  |
| XL-VLDL-FC_% |  |  |  |
| XL-VLDL-TG_% |  |  |  |
| L-VLDL-PL_% |  |  |  |
| L-VLDL-C_% |  |  |  |
| L-VLDL-CE_% |  |  |  |
| L-VLDL-FC_% |  |  |  |
| L-VLDL-TG_% |  |  |  |
| M-VLDL-PL_% |  |  |  |
| M-VLDL-C_% |  |  |  |
| M-VLDL-CE_% |  |  |  |
| M-VLDL-FC_% |  |  |  |
| M-VLDL-TG_% |  |  |  |
| S-VLDL-PL_% |  |  |  |
| S-VLDL-C_% |  |  |  |
| S-VLDL-CE_% |  |  |  |
| S-VLDL-FC_% |  |  |  |
| S-VLDL-TG_% |  |  |  |
| XS-VLDL-PL_% |  |  |  |
| XS-VLDL-C_% |  |  |  |
| XS-VLDL-CE_% |  |  |  |
| XS-VLDL-FC_% |  |  |  |
| XS-VLDL-TG_% |  |  |  |
| IDL-PL_% |  |  |  |
| IDL-C_% |  |  |  |
| IDL-CE_% |  |  |  |
| IDL-FC_% |  |  |  |
| IDL-TG_% |  |  |  |
| L-LDL-PL_% |  |  |  |
| L-LDL-C_% |  |  |  |
| L-LDL-CE_% |  |  |  |
| L-LDL-FC_% |  |  |  |
| L-LDL-TG_% |  |  |  |
| M-LDL-PL_% |  |  |  |
| M-LDL-C_% |  |  |  |
| M-LDL-CE_% |  |  |  |
| M-LDL-FC_% |  |  |  |
| M-LDL-TG_% |  |  |  |
| S-LDL-PL_% |  |  |  |
| S-LDL-C_% |  |  |  |
| S-LDL-CE_% |  |  |  |
| S-LDL-FC_% |  |  |  |
| S-LDL-TG_% |  |  |  |
| XL-HDL-PL_% |  |  |  |
| XL-HDL-C_% |  |  |  |
| XL-HDL-CE_% |  |  |  |
| XL-HDL-FC_% |  |  |  |
| XL-HDL-TG_% |  |  |  |
| L-HDL-PL_% |  |  |  |
| L-HDL-C_% |  |  |  |
| L-HDL-CE_% |  |  |  |
| L-HDL-FC_% |  |  |  |
| L-HDL-TG_% |  |  |  |
| M-HDL-PL_% |  |  |  |
| M-HDL-C_% |  |  |  |
| M-HDL-CE_% |  |  |  |
| M-HDL-FC_% |  |  |  |
| M-HDL-TG_% |  |  |  |
| S-HDL-PL_% |  |  |  |
| S-HDL-C_% |  |  |  |
| S-HDL-CE_% |  |  |  |
| S-HDL-FC_% |  |  |  |
| S-HDL-TG_% |  |  |  |
| VLDL-D |  |  |  |
| LDL-D |  |  |  |
| HDL-D |  |  |  |
| Serum-C |  |  |  |
| VLDL-C |  |  |  |
| Remnant-C |  |  |  |
| LDL-C |  |  |  |
| HDL-C |  |  |  |
| HDL2-C |  |  |  |
| HDL3-C |  |  |  |
| EstC |  |  |  |
| FreeC |  |  |  |
| Serum-TG |  |  |  |
| VLDL-TG |  |  |  |
| LDL-TG |  |  |  |
| HDL-TG |  |  |  |
| TotPG |  |  |  |
| TG/PG |  |  |  |
| PC |  |  |  |
| SM |  |  |  |
| TotCho |  |  |  |
| ApoA1 |  |  |  |
| ApoB |  |  |  |
| ApoB/ApoA1 |  |  |  |
| TotFA |  |  |  |
| UnSat |  |  |  |
| DHA |  |  |  |
| LA |  |  |  |
| FAn3 |  |  |  |
| FAn6 |  |  |  |
| PUFA |  |  |  |
| MUFA |  |  |  |
| SFA |  |  |  |
| DHA/FA |  |  |  |
| LA/FA |  |  |  |
| FAn3/FA |  |  |  |
| FAn6/FA |  |  |  |
| PUFA/FA |  |  |  |
| MUFA/FA |  |  |  |
| SFA/FA |  |  |  |
| Cit |  |  |  |
| Ala |  |  |  |
| Gln |  |  |  |
| His |  |  |  |
| Ile |  |  |  |
| Leu |  |  |  |
| Val |  |  |  |
| Phe |  |  |  |
| Tyr |  |  |  |
| Ace |  |  |  |
| AcAce |  |  |  |
| bOHBut |  |  |  |
| Crea |  |  |  |
| Alb |  |  |  |
| Gp |  |  |  |

Significance determined at p<0.05 and q<0.20. Beta-regression coefficients (standard errors) indicate change in servings of fruit and vegetable consumption per 1-standard deviation increase in log-transformed metabolite adjusted for age, gender and education.

**Supplementary Table 6.** Metabolites associated with alcohol consumption on a continuous scale

|  | ß (SE) | p-value | q-value |
| --- | --- | --- | --- |
| XXL-VLDL-P |  |  |  |
| XXL-VLDL-L |  |  |  |
| XXL-VLDL-PL |  |  |  |
| XXL-VLDL-C |  |  |  |
| XXL-VLDL-CE |  |  |  |
| XXL-VLDL-FC |  |  |  |
| XXL-VLDL-TG |  |  |  |
| XL-VLDL-P |  |  |  |
| XL-VLDL-L |  |  |  |
| XL-VLDL-PL |  |  |  |
| XL-VLDL-C |  |  |  |
| XL-VLDL-CE |  |  |  |
| XL-VLDL-FC |  |  |  |
| XL-VLDL-TG |  |  |  |
| L-VLDL-P |  |  |  |
| L-VLDL-L |  |  |  |
| L-VLDL-PL |  |  |  |
| L-VLDL-C |  |  |  |
| L-VLDL-CE |  |  |  |
| L-VLDL-FC |  |  |  |
| L-VLDL-TG |  |  |  |
| M-VLDL-P |  |  |  |
| M-VLDL-L |  |  |  |
| M-VLDL-PL |  |  |  |
| M-VLDL-C |  |  |  |
| M-VLDL-CE |  |  |  |
| M-VLDL-FC |  |  |  |
| M-VLDL-TG |  |  |  |
| S-VLDL-P |  |  |  |
| S-VLDL-L |  |  |  |
| S-VLDL-PL |  |  |  |
| S-VLDL-C |  |  |  |
| S-VLDL-CE |  |  |  |
| S-VLDL-FC |  |  |  |
| S-VLDL-TG |  |  |  |
| XS-VLDL-P |  |  |  |
| XS-VLDL-L |  |  |  |
| XS-VLDL-PL |  |  |  |
| XS-VLDL-C |  |  |  |
| XS-VLDL-CE |  |  |  |
| XS-VLDL-FC |  |  |  |
| XS-VLDL-TG |  |  |  |
| IDL-P |  |  |  |
| IDL-L |  |  |  |
| IDL-PL |  |  |  |
| IDL-C |  |  |  |
| IDL-CE |  |  |  |
| IDL-FC |  |  |  |
| IDL-TG |  |  |  |
| L-LDL-P |  |  |  |
| L-LDL-L |  |  |  |
| L-LDL-PL |  |  |  |
| L-LDL-C |  |  |  |
| L-LDL-CE |  |  |  |
| L-LDL-FC |  |  |  |
| L-LDL-TG |  |  |  |
| M-LDL-P |  |  |  |
| M-LDL-L |  |  |  |
| M-LDL-PL |  |  |  |
| M-LDL-C |  |  |  |
| M-LDL-CE |  |  |  |
| M-LDL-FC |  |  |  |
| M-LDL-TG |  |  |  |
| S-LDL-P |  |  |  |
| S-LDL-L |  |  |  |
| S-LDL-PL |  |  |  |
| S-LDL-C |  |  |  |
| S-LDL-CE |  |  |  |
| S-LDL-FC |  |  |  |
| S-LDL-TG |  |  |  |
| XL-HDL-P |  |  |  |
| XL-HDL-L |  |  |  |
| XL-HDL-PL |  |  |  |
| XL-HDL-C |  |  |  |
| XL-HDL-CE |  |  |  |
| XL-HDL-FC |  |  |  |
| XL-HDL-TG |  |  |  |
| L-HDL-P |  |  |  |
| L-HDL-L |  |  |  |
| L-HDL-PL |  |  |  |
| L-HDL-C |  |  |  |
| L-HDL-CE |  |  |  |
| L-HDL-FC |  |  |  |
| L-HDL-TG |  |  |  |
| M-HDL-P |  |  |  |
| M-HDL-L |  |  |  |
| M-HDL-PL |  |  |  |
| M-HDL-C |  |  |  |
| M-HDL-CE |  |  |  |
| M-HDL-FC |  |  |  |
| M-HDL-TG |  |  |  |
| S-HDL-P |  |  |  |
| S-HDL-L |  |  |  |
| S-HDL-PL |  |  |  |
| S-HDL-C |  |  |  |
| S-HDL-CE |  |  |  |
| S-HDL-FC |  |  |  |
| S-HDL-TG |  |  |  |
| XXL-VLDL-PL_% |  |  |  |
| XXL-VLDL-C_% |  |  |  |
| XXL-VLDL-CE_% |  |  |  |
| XXL-VLDL-FC_% |  |  |  |
| XXL-VLDL-TG_% |  |  |  |
| XL-VLDL-PL_% |  |  |  |
| XL-VLDL-C_% |  |  |  |
| XL-VLDL-CE_% |  |  |  |
| XL-VLDL-FC_% |  |  |  |
| XL-VLDL-TG_% |  |  |  |
| L-VLDL-PL_% |  |  |  |
| L-VLDL-C_% |  |  |  |
| L-VLDL-CE_% |  |  |  |
| L-VLDL-FC_% |  |  |  |
| L-VLDL-TG_% |  |  |  |
| M-VLDL-PL_% |  |  |  |
| M-VLDL-C_% |  |  |  |
| M-VLDL-CE_% |  |  |  |
| M-VLDL-FC_% |  |  |  |
| M-VLDL-TG_% |  |  |  |
| S-VLDL-PL_% |  |  |  |
| S-VLDL-C_% |  |  |  |
| S-VLDL-CE_% |  |  |  |
| S-VLDL-FC_% |  |  |  |
| S-VLDL-TG_% |  |  |  |
| XS-VLDL-PL_% |  |  |  |
| XS-VLDL-C_% |  |  |  |
| XS-VLDL-CE_% |  |  |  |
| XS-VLDL-FC_% |  |  |  |
| XS-VLDL-TG_% |  |  |  |
| IDL-PL_% |  |  |  |
| IDL-C_% |  |  |  |
| IDL-CE_% |  |  |  |
| IDL-FC_% |  |  |  |
| IDL-TG_% |  |  |  |
| L-LDL-PL_% |  |  |  |
| L-LDL-C_% |  |  |  |
| L-LDL-CE_% |  |  |  |
| L-LDL-FC_% |  |  |  |
| L-LDL-TG_% |  |  |  |
| M-LDL-PL_% |  |  |  |
| M-LDL-C_% |  |  |  |
| M-LDL-CE_% |  |  |  |
| M-LDL-FC_% |  |  |  |
| M-LDL-TG_% |  |  |  |
| S-LDL-PL_% |  |  |  |
| S-LDL-C_% |  |  |  |
| S-LDL-CE_% |  |  |  |
| S-LDL-FC_% |  |  |  |
| S-LDL-TG_% |  |  |  |
| XL-HDL-PL_% |  |  |  |
| XL-HDL-C_% |  |  |  |
| XL-HDL-CE_% |  |  |  |
| XL-HDL-FC_% |  |  |  |
| XL-HDL-TG_% |  |  |  |
| L-HDL-PL_% |  |  |  |
| L-HDL-C_% |  |  |  |
| L-HDL-CE_% |  |  |  |
| L-HDL-FC_% |  |  |  |
| L-HDL-TG_% |  |  |  |
| M-HDL-PL_% |  |  |  |
| M-HDL-C_% |  |  |  |
| M-HDL-CE_% |  |  |  |
| M-HDL-FC_% |  |  |  |
| M-HDL-TG_% |  |  |  |
| S-HDL-PL_% |  |  |  |
| S-HDL-C_% |  |  |  |
| S-HDL-CE_% |  |  |  |
| S-HDL-FC_% |  |  |  |
| S-HDL-TG_% |  |  |  |
| VLDL-D |  |  |  |
| LDL-D |  |  |  |
| HDL-D |  |  |  |
| Serum-C |  |  |  |
| VLDL-C |  |  |  |
| Remnant-C |  |  |  |
| LDL-C |  |  |  |
| HDL-C |  |  |  |
| HDL2-C |  |  |  |
| HDL3-C |  |  |  |
| EstC |  |  |  |
| FreeC |  |  |  |
| Serum-TG |  |  |  |
| VLDL-TG |  |  |  |
| LDL-TG |  |  |  |
| HDL-TG |  |  |  |
| TotPG |  |  |  |
| TG/PG |  |  |  |
| PC |  |  |  |
| SM |  |  |  |
| TotCho |  |  |  |
| ApoA1 |  |  |  |
| ApoB |  |  |  |
| ApoB/ApoA1 |  |  |  |
| TotFA |  |  |  |
| UnSat |  |  |  |
| DHA |  |  |  |
| LA |  |  |  |
| FAn3 |  |  |  |
| FAn6 |  |  |  |
| PUFA |  |  |  |
| MUFA |  |  |  |
| SFA |  |  |  |
| DHA/FA |  |  |  |
| LA/FA |  |  |  |
| FAn3/FA |  |  |  |
| FAn6/FA |  |  |  |
| PUFA/FA |  |  |  |
| MUFA/FA |  |  |  |
| SFA/FA |  |  |  |
| Cit |  |  |  |
| Ala |  |  |  |
| Gln |  |  |  |
| His |  |  |  |
| Ile |  |  |  |
| Leu |  |  |  |
| Val |  |  |  |
| Phe |  |  |  |
| Tyr |  |  |  |
| Ace |  |  |  |
| AcAce |  |  |  |
| bOHBut |  |  |  |
| Crea |  |  |  |
| Alb |  |  |  |
| Gp |  |  |  |

Significance determined at p<0.05 and q<0.20. Beta-regression coefficients (standard errors) indicate change in grams of alcohol per 1-standard deviation increase in log-transformed metabolite adjusted for age, gender and education.

**Supplementary Figure 1.** Parallel analysis scree plots for metabolite classes, a) amino acids, b) fatty acids, c) lipoprotein group 1 and d) lipoprotein group 2

**a)**


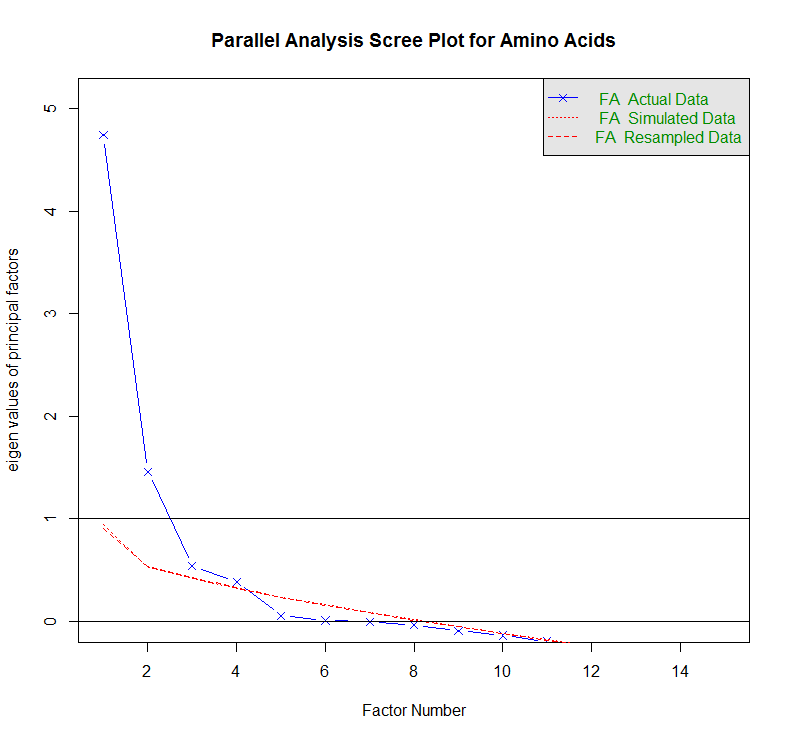


Factor number

Eigen values

Factor number


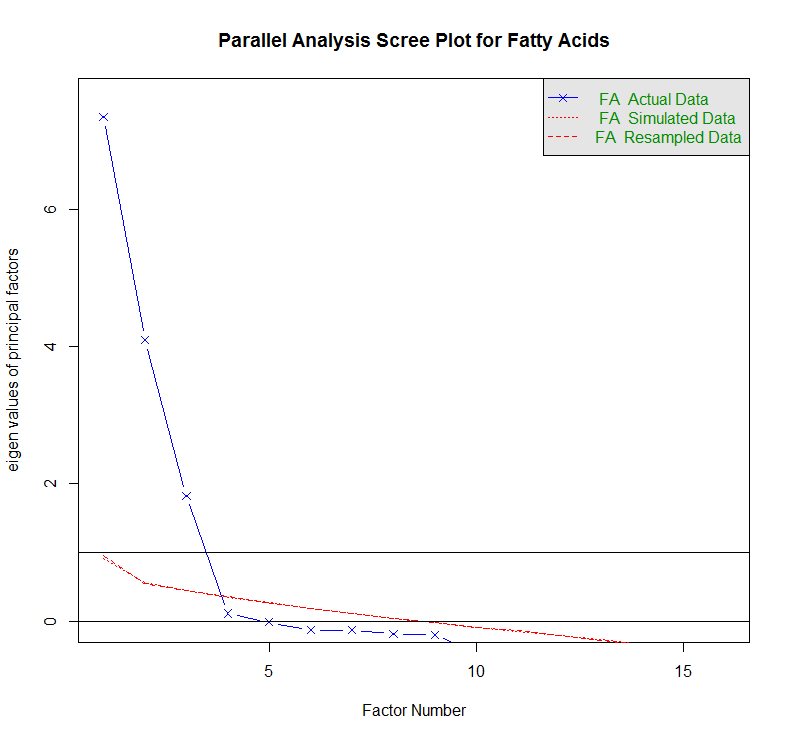
**b)**

Eigen values

Factor number


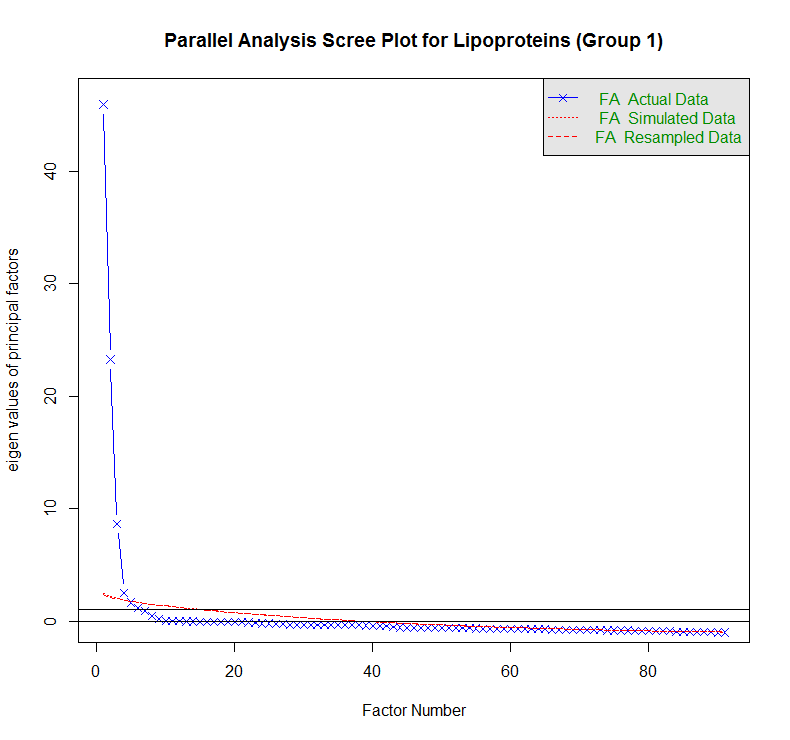
**c)**

Factor number

Eigen values

**d)**
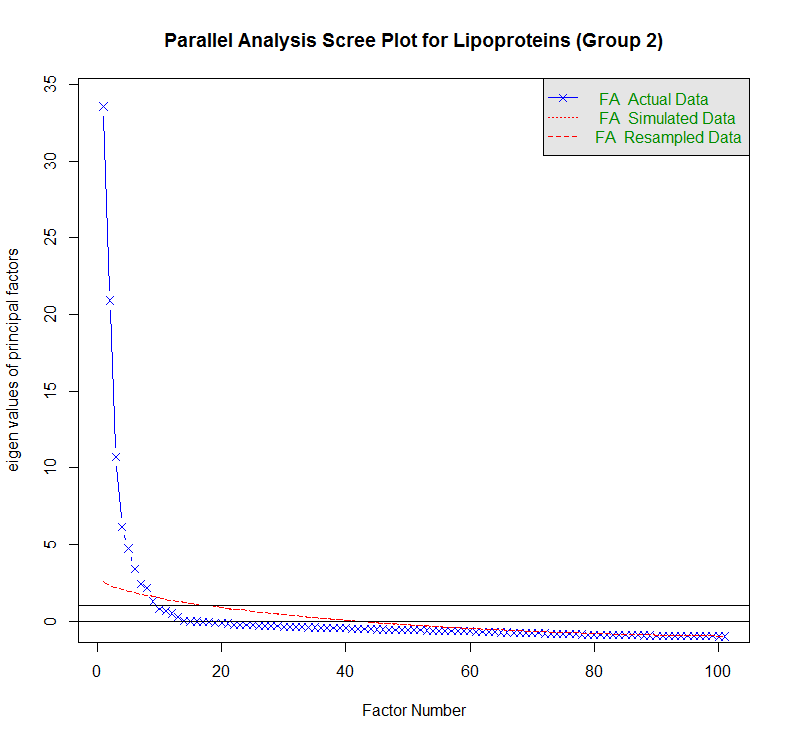


Eigen values

Factor number
